# Cancer Progression Gene Expression Profiling Identifies the Urokinase Plasminogen Activator Receptor as a Biomarker of Metastasis in Cutaneous Squamous Cell Carcinoma

**DOI:** 10.3389/fonc.2022.835929

**Published:** 2022-04-11

**Authors:** Elahe Minaei, Simon A. Mueller, Bruce Ashford, Amarinder Singh Thind, Jenny Mitchell, Jay R. Perry, Benjamin Genenger, Jonathan R. Clark, Ruta Gupta, Marie Ranson

**Affiliations:** ^1^ Illawarra Health and Medical Research Institute (IHMRI), Wollongong, NSW, Australia; ^2^ School of Chemistry and Molecular Bioscience, University of Wollongong, Wollongong, NSW, Australia; ^3^ Department of Head and Neck Surgery, Sydney Head and Neck Cancer Institute, Chris O’Brien Lifehouse, Sydney, NSW, Australia; ^4^ Department for Otorhinolaryngology, Head and Neck Surgery, Zurich University Hospital University of Zurich, Zurich, Switzerland; ^5^ Illawarra and Shoalhaven Local Health District (ISLHD), Wollongong, NSW, Australia; ^6^ School of Medicine, University of Wollongong, Wollongong, NSW, Australia; ^7^ Royal Prince Alfred Institute of Academic Surgery, Sydney Local Health District, Sydney, NSW, Australia; ^8^ Central Clinical School, Faculty of Medicine and Health, The University of Sydney, Sydney, NSW, Australia; ^9^ NSW Health Pathology, Department of Tissue Pathology and Diagnostic Oncology, Royal Prince Alfred Hospital, Sydney, NSW, Australia

**Keywords:** cutaneous squamous cell carcinoma (cSSC), urokinase plasminogen activator receptor (uPAR), urokinase plasminogen activator (uPA), metastasis, matrix metalloproteinase (MMP), extracellular matrix (ECM), tumor stroma, transcriptomics

## Abstract

Cutaneous squamous cell carcinoma (cSCC) of the head and neck region is the second most prevalent skin cancer, with metastases to regional lymph nodes occurring in 2%–5% of cases. To further our understanding of the molecular events characterizing cSCC invasion and metastasis, we conducted targeted cancer progression gene expression and pathway analysis in non-metastasizing (PRI-) and metastasizing primary (PRI+) cSCC tumors of the head and neck region, cognate lymph node metastases (MET), and matched sun-exposed skin (SES). The highest differentially expressed genes in metastatic (MET and PRI+) versus non-metastatic tumors (PRI-) and SES included *PLAU*, *PLAUR*, *MMP1*, *MMP10*, *MMP13*, *ITGA5*, *VEGFA*, and various inflammatory cytokine genes. Pathway enrichment analyses implicated these genes in cellular pathways and functions promoting matrix remodeling, cell survival and migration, and epithelial to mesenchymal transition, which were all significantly activated in metastatic compared to non-metastatic tumors (PRI-) and SES. We validated the overexpression of urokinase plasminogen activator receptor (uPAR, encoded by *PLAUR*) in an extended patient cohort by demonstrating higher uPAR staining intensity in metastasizing tumors. As pathway analyses identified epidermal growth factor (EGF) as a potential upstream regulator of *PLAUR*, the effect of EGF on uPAR expression levels and cell motility was functionally validated in human metastatic cSCC cells. In conclusion, we propose that uPAR is an important driver of metastasis in cSCC and represents a potential therapeutic target in this disease.

## 1 Introduction

Cutaneous squamous cell carcinoma (cSCC) is a prevalent non-melanoma skin cancer worldwide (1). As principally a disease of the sun-exposed skin, most notably in the head and neck, cSCC is particularly prevalent in regions with intensive sun exposure such as Australasia where it represents a significant health burden (2, 3). Metastasis to regional lymph nodes in the head neck occurs in <5% of cases but imparts significant morbidity and mortality (4). Notwithstanding conventional systemic treatment options such as chemotherapy and, more recently, epidermal growth factor receptor inhibitors or immunotherapy, for a substantial proportion of advanced cSCC patients there are still no valid second-line therapies (5), indicating a need for alternate targeted therapy options and strategies.

Conventional clinicopathologic prognostic markers in cSCC are unreliable predictors of lymph node metastasis (6–8). Recent studies assessing the genomic and transcriptomic landscape of cSCC have revealed heterogeneity of cellular subtypes in these cancers; however, tumor cell populations harboring potentially clinically useful gene signatures and/or therapeutic targets of metastatic risk in primary cSCC are evident (1, 9–16). These biomarkers or molecular signatures of invasion and metastasis are overwhelmingly related to cancer progression pathways encompassing extracellular matrix (ECM) interactions and remodeling, epithelial to mesenchymal transition (EMT), cellular motility, and survival.

Altered proteolysis and EMT programs are required for ECM remodeling and tumor cell escape (17–19). In particular, overexpression of the urokinase plasminogen activator (uPA, encoded by *PLAU*) and its cognate cell surface receptor (uPAR, encoded by *PLAUR*) (including downstream effector and upstream regulator molecules) is associated with EMT (20) and correlates with increased metastasis and/or poorer patient survival in many solid tumor types (21–23) including mucosal squamous cell carcinoma of the oral cavity (24–30).

These genes and their proteins are also overexpressed in advanced and metastatic cSCC (15, 31, 32) with uPAR mRNA shown to be localized to a subpopulation of invasive cells in primary cSCCs (33). Upon binding to uPAR, uPA efficiently activates co-localized plasminogen to the potent broad-spectrum protease plasmin, which initiates a cascade of pericellular proteolysis that directly and indirectly (through the activation of pro-metalloproteinases, pro-MMPs) degrades integral ECM molecules including fibronectin, laminins, elastins, and collagens, thus enabling tumor cell invasion and dissemination (18, 22). Plasmin and MMPs are also responsible for the release and activation of latent growth/angiogenic factors (such as EGF and VEGF) and chemokines from the ECM, which promotes cellular proliferation, survival, and motility (18, 22). Activated receptor tyrosine kinase pathways have also been shown to enhance uPA system expression in cancer (23).

While others have either specifically or coincidently explored the expression of the uPA system, MMPs, and ECM interactors in cSCC (summarized in **Table S1**), few have focused exclusively on the uPA system in UV-induced cSCC of the head and neck encompassing the spectrum of disease states. To this end, we performed gene expression analyses using a curated cancer progression-targeted gene set in non-metastasizing and metastasizing head and neck cSCC primary tumors, lymph node metastases, and matched sun-exposed skin (SES). This was then used for gene enrichment and pathway analyses. An integrated gene expression was also performed on relevant gene expression omnibus (GEO) datasets to strengthen our findings. Recognizing the *PLAUR* gene as an important mediator of proteolytic networks in the tumor microenvironment, we further investigated uPAR protein levels and association with metastatic disease. Finally, the predicted activating effects of EGF was assessed *in vitro* on EGFR-expressing human metastatic cSCC cell lines.

## 2 Material and Methods

### 2.1 Study Population and Sample Collection

The project was approved by the University of Wollongong Health and Medical Human Research Ethics Committee (Wollongong NSW, Australia, UOW/ISLHD HREC 14/397). Head and neck cSCC specimens from a total of 50 patients who underwent surgery with curative intent were retrieved from the Department of Tissue Pathology and Diagnostic Oncology at Royal Prince Alfred Hospital, Sydney NSW, Australia. Formalin-fixed paraffin-embedded (FFPE) specimens were derived from the head and neck region of 21 patients with primary tumors with no evidence of metastasis (PRI-), 14 patients with primary tumors that had metastasized (PRI+) (13 of which had available concurrent metastases), and an additional 15 patients with lymph node metastases, but with no available primary tumor. FFPE cores from SES were taken from the peripheral negative margins where available. The specimens used are summarized in **Supplementary Data Sheet 1**. High-risk disease was defined as per criteria of the 7th edition of the American Joint Commission on Cancer Staging Manual (34). Patients in the non-metastatic group had to meet one or more of the following criteria: absence of metastases at the >24-month follow-up after resection of the primary; negative sentinel lymph node biopsy at the time of resection of the primary; or histologically negative prophylactic neck dissection. Clinical features, treatment, and follow-up were obtained from the Sydney Head and Neck Cancer Institute database. For comparisons between the cohorts, the Mann–Whitney-U test was applied for non-parametric continuous data, the Fisher’s exact for categorical data in 2 × 2 contingency tables, and χ^2^ test for larger contingency tables.

### 2.2 RNA Extraction

Specimens underwent histopathological review to select areas with high neoplastic content (>30%) and exclude areas containing necrosis, hemorrhage, high keratin content, or significant inflammation. Three to six tissue cores (2 mm diameter) were then obtained from FFPE blocks of these specimens for deparaffinization and homogenization prior to RNA extraction. Tumor nucleic acids from specimens were extracted using AllPrep DNA/RNA FFPE Kit (80234, Qiagen, Hilden, Germany) according to the manufacturer’s instructions. RNA samples that met initial QC measures including high A260/280 (<1.8–2) and acceptable integrity (Invitrogen Qubit RNA IQ Assay, Thermo Fisher Scientific, Waltham, MA, USA) were utilized in gene expression assays.

### 2.3 Gene Expression Assays and Data Analysis

Up to 150 ng of purified RNA was run on the nCounter Sprint (NanoString Technologies, Seattle, WA, USA) platform using the nCounter PanCancer Progression Panel (NanoString; 740 target genes, 30 housekeeping genes) as per the manufacturer’s instructions. nSolver Analysis Software 4.0 (NanoString) was used to remove specimens with low binding density or other technical QC flags. The raw data from the remaining specimens was then processed using the iterative RUVSeq normalization pipeline for QC, normalization, and data visualization/validation using NanoNormIter R package (35). After technical quality control steps, specimens SESP3, SESP29, and PRI+P7 were excluded from further analysis because of very low geometric mean of housekeeping gene expression. Housekeeping genes associated with phenotype were also excluded using the glm.nb function (Negative Binomial Generalized Linear Model) as specified in the RUVseq-based pipeline [refer to (35)]. The normalization step of all possible combinations of pairwise analyses was tested using different values of *k* (RUVg) and the different normalized expression datasets visualized using principal component analysis (PCA) and RLE plots to detect problematic samples for assessment of removal from further analysis. By this method, METP16 was flagged, assessed, and discarded. The final list of included specimens that underwent NanoString analyses are shown in **Supplementary Data Sheet 1**. After RUVg normalization of final specimens, differential expression analyses were performed using DESeq2. The top differentially expressed genes (DEGs) were selected based on both log_2_fold change between the compared groups and the *p*-values adjusted for multiple testing using the Benjamini–Hochberg method (36). **Supplementary Data Sheet 2** contains differential gene expression data for all cohort comparisons.

Where indicated, raw data from the retained specimens that passed these QC steps were also analyzed using the global significance score function within the nCounter Advanced Analysis 2.0 software (NanoString) which is derived using the most DEGs in gene sets representative of a particular cancer progression annotation.

### 2.4 Functional Enrichment Analysis

Ingenuity Pathway Analysis (IPA; Qiagen Inc., https://www.qiagenbioinformatics.com/products/ingenuitypathway-analysis) software was used to generate networks and functional analyses of gene expression datasets (37). IPA core analysis default settings were used, limited to the human knowledge base. We applied a global molecular network developed from information contained in the Ingenuity Pathways Knowledge Base incorporating DEGs from our study with log2 fold change (logFC) <-0.58, >0.58 (*p*-value < 0.05) for each comparison. Networks of these gene lists were then generated algorithmically based on their interrelationships. The significance of the association between lists of DEGs and the Diseases and Functions were assessed using (1) the ratio of DEGs (molecules) from the dataset that map to a specific cellular and molecular function category (in relation to the total number of molecules included in the particular disease and function) and (2) Fischer’s exact test (to determine the likelihood of association between the molecules in the dataset and the disease and function).

IPA uses the activation z-score algorithm to make a prediction of activation or inhibition (or no prediction) as well as to reduce the chance that random data will generate significant predictions. Causal Network and Upstream Regulator analyses were used to identify regulators with a probability of being responsible for the changes in gene expression observed, by calculating an overlap p-value with Fisher’s exact test and an activation z-score. Causal Networks are small hierarchical networks of regulators that control the expression of the dataset targets.

### 2.5 Integrative Gene Expression Meta-Analysis Using the Robust Rank Aggregation Approach

An expression meta-analysis study was performed on all available cSCC datasets in Gene Expression Omnibus (GEO) (38) containing normal skin from sun-exposed areas and cSCC cases classified as invasive or metastatic for comparison to our PRI+ vs. SES analyses. Using the detailed filtering criteria described in **Table S2**, only three datasets comprising 18 SES and 25 cSCC samples matched these criteria. In the first step, three separate differential expression analyses for each dataset was performed using the Limma (39) and GEOquery (40) packages. A universal threshold of *p*-value < 0.01 and logFC <-0.58, >0.58 was used for the collection of significantly DEG lists for each comparison. RankerGUI (41) ranked the DEG lists based on the logFC values, which were then used for a differential meta-analysis using the Robust Rank Aggregation (RRA) method (42). Significant DEG lists of the meta-analysis were extracted using a *p-*value cutoff < 0.05. In addition, Reactome (43) functional enrichment analysis of significantly DEG was carried out using Bioconductor package-ReactomePA (44).

### 2.6 Immunohistochemistry

Immunohistochemical staining for uPAR was performed using FFPE tissues from primary and metastatic cSCC specimens (listed in **Supplementary Data Sheet 1**). Briefly, 4-µm sections were deparaffinized and uPAR detected (after antigen retrieval at 100°C in pH 9.0 solution) with anti-uPAR at either 1:100 dilution (clone R4; Dako, Glostrup, Denmark) or 1:500 (10925-T30; Sino Biological, Chesterbrook, PA, USA) using the Ventana BenchMark Ultra Automated Immunohistochemistry (IHC)/ISH slide staining system with diaminobenzidine (DAB) as chromogen, followed by counterstaining with hematoxylin. The confounder effect of using the two different sources of anti-uPAR in this study was not significant (data not shown). Slides containing neutrophils and macrophages as internal and external positive controls, respectively, accompanied all staining runs. The proportion of tumor cells demonstrating complete membranous staining with uPAR was initially recorded as a proportion of the total number of tumor cells at the advancing edge of the tumor. Complete membranous staining of any intensity of the tumor cells was then scored and used for statistical analyses in this study. Scores were analyzed in GraphPad Prism 8.4.3.

### 2.7 miRNA Analysis

Small RNA-Seq was performed using the Illumina HiSeq platform at the Australian Genome Research Facility Ltd., Westmead, NSW, Australia. The quality test of raw reads was assessed using the FastQC tool v0.11.9 (https://www.bioinformatics.babraham.ac.uk/projects/fastqc/). Poor-quality reads were trimmed using Cutadapt (version 2.8). Trimmed fastq sequences were mapped and annotated using sRNAbench (45). Next, a differential expression analysis based on negative binomial distribution was performed using the sRNAde tool (46), which integrates Deseq2 (47) and EdgeR (48). Further, significantly differentially expressed miRNAs were extracted based on log_2_FC ≥ ± 1 and *p*-values adjusted for multiple testing using the Benjamini–Hochberg method (36). In downstream analyses, miRDB (49, 50) was used to obtain putative targeted genes of statistically significant miRNAs. miRDB provides a collection of miRNA and mRNA interactions predicted by the Machine Learning Tool (MirTarget), which utilizes features related to miRNA binding and downregulated targets. miRNA–mRNA interactions having a score >75 were considered for further analysis. Finally, experimentally validated miRNA–mRNA interactions for *PLAUR* from the miRtarbase database (51) were explored. Two-tailed Spearman correlation coefficient between uPAR IHC and miRNA was calculated using GraphPad Prism 9.0.2.

### 2.8 Cell-Based Assays

The effect of EGF on cell migration was assessed in a scratch-wound assay using the IncuCyte^®^ Zoom Kinetic Imaging System (Essen BioScience, Ann Arbor, MI, USA). Patient-derived metastatic cSCC cell line UW-CSCC2 [described in detail in (52)] was seeded onto collagen 1-coated 96-well ImageLock plates (Essen). After 24 h incubation in low serum containing media (DMEM supplemented with 1% FCS, no EGF), the cells were scratched according to manufacturer’s instructions using the 96-pin Essen Woundmaker™. The cells were subsequently washed with serum-free media, then incubated with 0, 5, 10, or 20 ng/ml human EGF ± 1 µM gefitinib in low serum media at 37°C, 5% CO_2_, and imaged over 24 h at ×10 objective to track cell motility and wound width. IncuCyte™ ZOOM software was used to analyze wound width reduction over time. Data were analyzed using GraphPad Prism 9.0.2.

For determination of uPAR levels, UW-CSCC2 cells were treated as above except that cells were lysed for total protein extraction and Western blotting 24 h after EGF ± 1 µM gefitinib treatment. Blots were incubated with anti-human uPAR rabbit polyclonal antibody (1:2,000; ab103791, Abcam) or anti-GAPDH mouse monoclonal antibody (1:5,000; G8795, Sigma-Aldrich, St. Louis, MO, USA) and detected using horseradish peroxidase-conjugated anti-rabbit IgG (7074S, Cell Signaling, Danvers, MA, USA) or anti-mouse IgG (ab205719, Abcam, Cambridge, MA, USA) both at 1:5,000 dilution. Chemiluminescence was generated using Pierce ECL Western Blotting Substrate (Thermo Fisher Scientific, Waltham, MA, USA) and visualized using a ChemiDoc MP Imaging System (Bio-Rad Laboratories). Densitometry was conducted using ImageJ (v1.53e, NIH, USA) and values normalized against the housekeeping protein GAPDH as protein loading control.

For detection of EGFR, cells were seeded into ibidi chamber slides (ibidi GmbH, Gräfelfing, Germany) and grown under regular culture conditions prior to staining with human anti-EGFR monoclonal antibody (1:1,000; MAB1095-100—R&D Systems, Minneapolis, MN, USA) followed by Alexa Fluor^®^ 555-conjugated donkey anti-mouse IgG H&L (1:2,000; ab150106, Abcam). The cells were then counterstained with ActinRed 555 ready probes (Thermo Fisher) and RedDot2 Far-Red Nuclear Stain (Biotium, Inc., Fremont, CA, USA), and then imaged with a ×20 oil immersion objective and a TCS SP5 confocal microscope (Leica, Wetzlar, Germany).

## 3 Results

### 3.1 Clinical and Demographic Characteristics

Clinical and demographic data are shown in **Table 1**. While the sex distribution was similar, patients suffering from metastasizing cSCC (PRI+ and/or MET) were significantly older than patients with non-metastasizing cSCC (PRI-; *p* = 0.034). This age difference may be subject to bias, since in patients with MET where the primary tumor was not known, the age was recorded at the time of treatment of the lymph node metastasis, which is at a later point of the course of the disease. The two groups differed significantly in TNM tumor stage at the time of surgery, which was expected since the presence of lymph node metastasis is the determinant of the N-stage (*p* < 0.001) and reflects in higher overall stage (*p* < 0.001). The validity of the difference in the T-stage is limited, since the primary was no longer present at the time of surgery in 12 MET samples and could not be retrospectively determined. Although not statistically significant, lympho-vascular infiltration (LVI) was more commonly seen in the metastatic cohort, which could be expected since LVI is a crucial step in the development of lymph node metastasis. The rate of perineural infiltration and histopathological grading did not significantly differ between the two groups. Full clinico-pathological data for each sample are listed in **Supplementary Data Sheet 1**.

**Table 1:** Demographic and clinical data of the cohort of 50 patients with cSCC with (cohorts PRI+, MET) or without lymph node metastasis (PRI-).

Variable	PRI+/MET (metastasizing tumors), n = 29	PRI- (locally confined tumors), n = 21	Total (n = 50)	p-value
Mean age, years (range)	74.8 (32 to 93)	68.2 (39 to 92)	72.1 (32 to 93)	0.034^c^
Sex, n (%)				
Female	2 (7)	4 (19)	6 (12)	0.22 *^d^*
Male	27 (93)	17 (81)	44 (88)	
Site of primary tumor, n (%)				
Scalp	4 (14)	5 (24)	9 (18)	0.99e *^e^*
Ear and temple	5 (17)	7 (33)	12 (24)	
Nose and midface	3 (10)	5 (24)	8 (16)	
Lip	2 (7)	2 (10)	4 (8)	
Neck	1 (3)	2 (10)	3 (6)	
Unknown	14 (48)	0	14 (28)
Recurrent tumor, n (%) *^a^*				
No	15 (52)	15 (71)	30 (60)	0.24 *^d^*
Yes	14 (48)	6 (29)	20 (40)	
T-stage at surgery, n (%) *^b^*				
0 or unknown	12 (41)	1 (5)	13 (26)	0.039 *^e^*
1	3 (10)	3 (14)	6 (12)	
2	6 (21)	4 (19)	10 (20)	
3	6 (21)	11 (52)	17 (34)	
4	2 (7)	2 (10)	4 (8)	
N-stage at surgery, n (%) *^b^*				
0	5 (17)	21 (100)	26 (52)	<0.001 *^e^*
1	5 (17)	0	5 (10)	
2	4 (14)	0	4 (8)	
3	13 (45)	0	13 (26)	
Unknown	2 (7)	0	2 (4)
Overall stage (AJCC 7th edition) *^b^*				
I	1 (3)	3 (14)	4 (8)	<0.001 *^e^*
II	2 (7)	4 (19)	6 (12)	
III	5 (17)	11 (52)	16 (32)	
IV	20 (69)	2 (10)	22 (44)	
Unknown	1 (3)	1 (3)	2 (4)
Histopathological grading, n (%) *^b^*				
1 (well differentiated)	2 (7)	2 (10)	4 (8)	0.13 *^e^*
2 (moderately differentiated)	13 (45)	14 (67)	27 (54)	
3 (poorly differentiated)	14 (48)	4 (19)	18 (36)	
Unknown	0	1 (5)	1 (2)
Lymph-vascular infiltration (LVI), n (%) *^b^*				
No	18 (62)	18 (86)	36 (72)	0.11 *^d^*
Yes	10 (34)	3 (14)	13 (26)	
Unknown	1 (3)	0	1 (2)
Perineural invasion (PNI), n (%) *^b^*				
No	11 (38)	11 (52)	22 (44)	0.56 *^d^*
Yes	15 (52)	10 (48)	25 (50)	
Unknown	3 (10)	0	3 (6)

a Recurrent tumors at surgery. Recurrences after last surgery are not included.

b When multiple samples of a single patient from the primary (PRI+) and lymph node metastasis (MET) were analyzed, the index tumor (PRI+) was prioritized.

c Mann–Whitney U test.

d Fisher exact test.

e Chi-square test.

### 3.2 Cancer Progression Pathways Involving ECM Remodeling and Cell Movement are Upregulated in a Stepwise Manner From SES to Non-Metastatic to Metastatic cSCC

#### 3.2.1 Gene Expression Analyses in Tumors Versus SES

**Figure 1:**
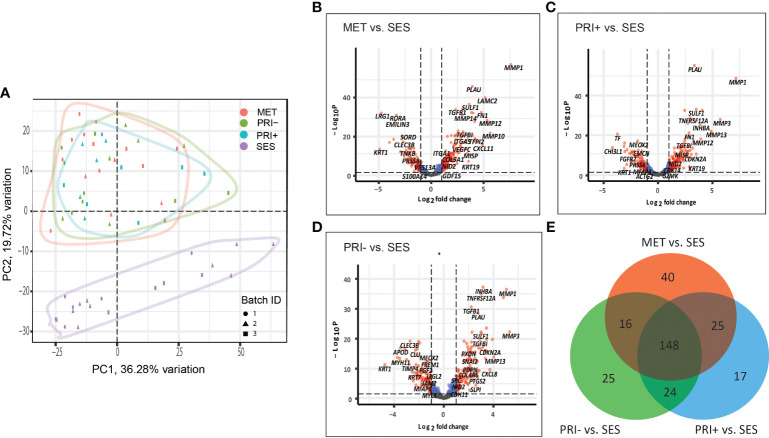
Cancer progression gene expression patterns of sun-exposed skin (SES) and non-metastatic (PRI-) and metastatic (PRI+ and MET) cSCC. **(A)** Principle component plot of tumor cohorts and SES normalized gene expression data. The first and second principal components are plotted on the x- and y-axis, respectively. Batch ID symbols indicate different NanoString runs and show lack of batch effect. **(B–D)** Volcano plots illustrating gene expression differences (x-axis) and significance (y-axis) (dotted horizontal lines) of **(B)** MET vs. SES, **(C)** PRI+ vs. SES, and **(D)** PRI- vs. SES. Each dot represents a gene. **(E)** Venn diagram depicting DEGs with log2 fold changes of ≥1 or ≤-1 between MET vs. SES, PRI+ vs. SES, and PRI- vs. SES and adjusted *p*-value ≤ 0.05. The number of DEGs for each pairwise comparison is indicated in the circles of the Venn diagram. The overlap between the circles shows DEGs that occur in more than one comparison.

A principal component analysis (PCA) plot based on all the normalized data of all cohort comparisons clearly separate the tumor cohorts (MET, PRI+, and PRI-) from SES (**Figure 1A**), with significant differential gene expression (log2FC ≥1 or ≤-1; adj*p*-value < 0.05) between all tumor cohorts and SES (**Figures 1B–D**). This included 229 DEGs in MET tumors vs. SES (147 up- and 82 downregulated), 214 in PRI+ (metastasizing primary tumors) vs. SES (133 up- and 81 downregulated), and 213 in PRI- (non-metastasizing primary tumors) vs. SES (124 up- and 89 downregulated) (refer to **Supplementary Data Sheet 2** for gene list). This highlights the striking differential gene expression in cSCC compared to SES despite the high mutational burden reported in SES (53). Fifty percent (148/295) of the DEGs between the three comparisons (i.e., between MET or PRI+ or PRI- vs. SES) were shared (**Figure 1E**; **Supplementary Data Sheet 2**). Of the top 20 upregulated shared DEGs (**Supplementary Data Sheet 2**), 12 are associated with MMP remodeling, cell motility, and ECM receptor interaction annotations (**Supplementary Image 1A**), indicating that these pathways are already dysregulated in non-metastasizing primary tumors. However, key MMP remodeling-associated genes *PLAU* and *MMP10* and basal cell marker *KRT19* were uniquely shared upregulated genes in metastatic tumors (MET and PRI+) (**Supplementary Image 1A**). Of the top 20 downregulated DEGs, 10 genes with varied functions such as keratinocyte differentiation (*KRT1*) and dysregulated tumor–microenvironment interactions were shared between all tumor cohorts and SES (**Supplementary Image 1B**). Four genes with disparate functions were among the top 20 downregulated genes uniquely shared by metastatic tumors compared to SES (**Supplementary Image 1B**). These unique shared up- and downregulated genes clustered the MET/PRI+ cohort together away from the PRI- cohort, which showed intermediate behavior between the metastatic tumors and SES (**Supplementary Image 1C**).

#### 3.2.2 Pathway Analyses of Gene Expression Profiles in Tumors Versus SES

**Figure 2:**
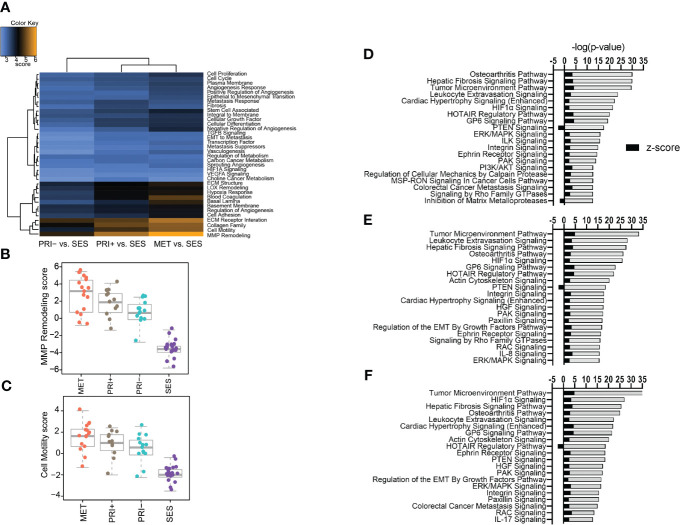
Pathway analyses of tumors vs. SES. **(A)** Heatmap of global significance scores of cancer progression gene annotations generated using nSolver Advanced Analysis software 2.0 (orange denotes gene sets whose genes exhibit extensive differential expression with the covariate (SES), blue denotes less differential expression). **(B, C)** Boxplots showing pathway scores (y-axis; fit using the first principal component of each gene set’s data) for two of the top differentially expressed cancer pathways specified in the heatmap. **(D–F)** Top 20 activated and inhibited canonical pathways (refer to **Supplementary Data File 3**) for **(D)** MET vs. SES, **(E)** PRI+ vs. SES, and **(F)** PRI- vs. SES showing significance level (-log (p-value)) along the x-axis and absolute activation z-score (<-2, >2) along the y-axis.

A gene set analysis of the differential gene expression profiles of tumor cohorts compared to SES confirmed that MMP remodeling followed by cell motility, collagen family, and ECM receptor interaction was the most differentially expressed cancer progression pathway (**Figure 2A**). Stepwise increases in expression from SES to PRI- to PRI+ and MET were most evident for MMP remodeling (**Figure 2B**) and cell motility (**Figure 2C**) annotations.

Ingenuity pathway analysis was then used to categorize the DEGs from the MET vs. SES, PRI+ vs. SES and PRI- vs. SES comparisons into canonical pathways. Significantly enriched canonical pathways [-log(p-value) >1.3, absolute value z-score >2, <-2] and the DEGs in each pathway are listed in **Supplementary Data Sheet 3**. In all tumor cohorts vs. SES, tumor microenvironment, leukocyte extravasation, hepatic fibrosis signaling pathway, and HIF1α signaling were among the top significantly activated CPs (**Figures 2D–F**). Significantly inhibited pathways included PTEN signaling (MET/PRI+ vs. SES only) and inhibition of matrix metalloproteinase (MET vs. SES only) (**Figure 2F**). Activation of leukocyte extravasation, which is the movement of leukocytes from the circulatory system toward a tumor [63], is in line with upregulated cell motility in tumors vs. SES found in our gene set analyses (**Figure 2C**). In line with other reports comparing cSCC vs. normal sun-exposed skin [64], ECM receptor interaction and interleukin signaling and PI3K/AKT/mTOR signaling were also significantly activated canonical pathways in our analyses (**Figures 2D–F**).

Integrative gene expression meta-analysis of publicly available invasive/metastatic cSCC (n = 25) vs. normal skin from sun-exposed area (n = 18) array data on the GEO platform (see **Table S2** for detailed sample filtering criteria) revealed a total of 127 upregulated and 59 downregulated significant DEGs (**Supplementary Data Sheet 4**). Comparison of these significantly DEGs with our PRI+ vs. SES dataset found 33 DEGs genes in common (**Supplementary Image 2A**). Reactome pathway analysis using these shared genes again highlight enrichment of activated pathways affecting extracellular matrix interactions, organization or degradation, collagen family, and interleukin/chemokine signaling (**Supplementary Image 2B**) as per our independent analyses using nSolver and IPA.

### 3.3 Differential Gene Expression and Pathway Analysis Between Metastatic and Non-Metastatic cSCCs Identifies *VEGFA*, *EGF*, and *IL1RN* as Key Upstream Regulators of Metastasis

#### 3.3.1 Gene Expression Analyses in Metastatic Versus Non-Metastatic Tumors

**Figure 3:**
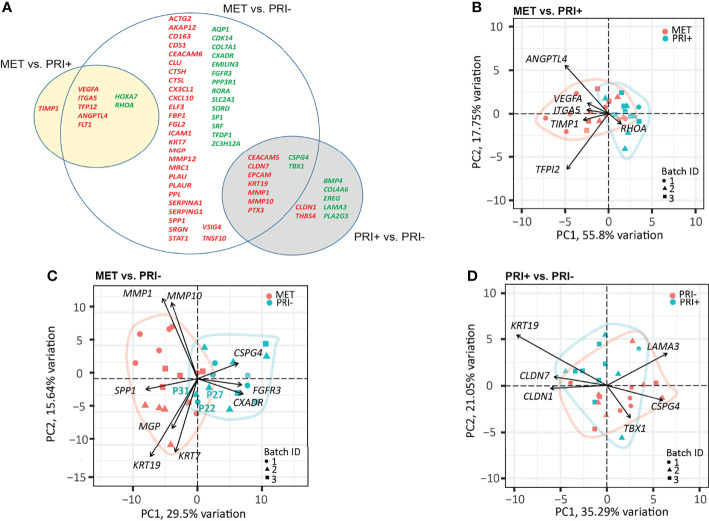
Cancer progression gene panel differential expression analysis between tumor cohorts. **(A)** Venn diagram depicting DEGs with log2 fold change ≥1 or ≤-1 between MET vs. PRI+ and MET vs. PRI- (adjusted *p*-values <0.05), and PRI+ vs. PRI- (*p*-values <0.01). Upregulated (red) and downregulated (green) DEGs for each pairwise comparison are indicated. The overlap between the circles show DEGs genes that occur in more than one comparison. **(B–D)** PCA loading plots based on significant DEGs between **(B)** MET and PRI+ (8), **(C)** MET and PRI- (54), and **(D)** PRI+ and PRI (16). Each symbol corresponds to one sample. Ellipses represent the region where the majority of samples are expected to fall. Non-overlapping ellipses imply that gene expression profiles cluster groups apart based on their distinct principal component scores. Batch ID symbols indicate samples analyzed in different NanoString runs and show lack of batch effect.

A progressive decrease in the number of significantly DEGs was found between the metastatic (PRI+) compared to non-metastatic (PRI-) tumors vs. MET (**Figure 3A**). The MET vs. PRI+ comparison revealed 8 significant DEGs while MET vs. PRI- revealed 58 significant DEGs (**Figure 3A**; **Supplementary Data Sheet 2**). At these stringent cutoffs, only 3 DEGs were found in the PRI+ vs. PRI- comparison (**Supplementary Data Sheet 2**) likely due to small sample size and bulk sampling (discussed further below). Using a less stringent cutoff for PRI+ vs. PRI- (*p* < 0.01 instead of adjp < 0.05), there were 16 significant DEGs (**Figure 3A**, gray circle). That there were few significant cancer progression gene expression differences between metastatic primaries and metastases suggested that the PRI+ tumors had acquired many of the activated pathways necessary for metastasis. In further support of this, pathway analysis of the six available patients’ specimens with matched MET, PRI+, and SES samples showed that the tumor pairs by and large clustered together and away from SES (**Supplementary Image 3**).

Of the 8 significantly DEGs between MET and PRI+, only one (*TIMP1*) was not shared with the MET vs. PRI- grouped cohort comparison (**Figure 3A**). Although *TIMP1* encodes an inhibitor of MMPs, elevated expression of TIMP1 has been reported in head and neck SCCs (54–60) and has been shown to stimulate cell proliferation and prevent apoptosis (61). A PCA loading plot using these 8 significant DEGs (**Figure 3B**) indicates that *ANGPTL4* (encodes angiopoietin) and *TFPI2* (encodes tissue factor inhibitor 2) exert the largest effects on PC1 and PC2 (followed by *VEGFA*, *ITGA5*, *TIMP1*, and *RHOA*). These genes are involved in various functions that promote either angiogenesis, cell adhesions or motility, protection from anoikis, matrix remodeling, or epithelial–mesenchymal transition (EMT) and are known to play important roles in the metastatic process in several cancers (62). Interestingly, *ITGA5*, which is also a classic EMT marker enriched on tumor-specific keratinocyte (TSK) subsets of metastatic cSCC (9) and upregulated in various cancers (63), showed a stepwise increase in expression from SES to PRI, to PRI+ to MET (**Supplementary Image 4**).

Although the MET and PRI- cohorts could be separated using the 58 significant DEGs from the MET vs. PRI- comparison, a few PRI- specimens (P22 and P31 in particular) clustered with the MET cohort (**Figure 3C**). A closer examination of the clinicopathological characteristics of these specimens found these to be from patients with high-risk features such as either recurrences or PNI. PCA loadings show that the genes exerting the largest effects include *MMP1* and *MMP10*, *KRT7*, and *KRT19* (**Figure 3C**), high levels of which in other cancers have been associated with unfavorable prognosis (64). Another example is *SPP1*, which encodes a stromal cell ligand shown to interact with integrin receptors encoded by *ITGB1* and *ITGA5*, which are both enriched on TSKs (9).

Of the 16 significant DEGs between PRI+ vs. PRI-, the majority were shared with the MET vs. PRI- comparison (**Figure 3A**). These genes feature MMPs (*MMP10*), cell differentiation and adhesion markers (*KRT19*, *CEACAM5*), and cell polarity and signal transducers (*CLND7*). While these genes exerted the largest effects on PRI+ as assessed by PCA loadings (**Figure 3D**), these cohorts were not distinguishable, possibly due to intra-tumoral heterogeneity and/or the particular area of primary tumor sampled.

#### 3.3.2 Pathway and Functional Analyses in Metastatic Versus Non-Metastatic Tumors

To further investigate the molecular mechanisms underlying cSCC progression, the IPA downstream effect analysis function was used to identify diseases and function activation status, given the observed differential gene expression data described above. A relatively small number of significantly activated functions (*p*-value > 9.89E-10, z-score > 2) were evident in MET vs. PRI+, and these were broadly associated with cellular movement (**Supplementary Image 5A** **;** **Supplementary Data Sheet 5**). Notably, functions associated with cell death and survival were either significantly decreased or inactivated (e.g., apoptosis/anoikis of tumor cell lines) or activated (e.g., cell viability). A larger number of significantly activated functions were found in MET vs. PRI- (**Supplementary Image 5B** **;** **Supplementary Data Sheet 5**) with top-scoring functional categories most strongly associated with cellular movement (inclusive of invasion/migration of cells, leucocyte migration, chemotaxis) and cell-to-cell signaling and interaction. Cellular movement was the main functional category predicted to be activated in the PRI+ vs. PRI- comparison (inclusive of migration of keratinocytes and fibroblasts); however, functions related to inflammatory response was the top activated category (**Figure 5C** **;** **Supplementary Data Sheet 5**).

**Figure 4:**
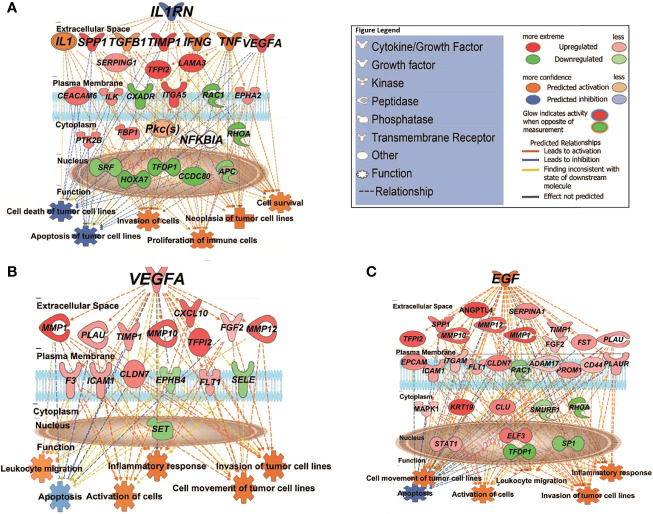
IPA causal network analysis depicting the interactions between upstream regulators, downstream genes, and physiological functions in cSCC. **(A)** MET vs. PRI+ comparison showing effect of predicted master regulator *IL1RN* (depth: 2). **(B, C)** MET vs. PRI- comparison showing predicted master regulators *VEGFA* (depth: 1) and *EGF* (depth: 1). Master regulators were predicted based on the causal paths known to influence the expression of their target genes leading to the physiological functions shown. Regulators with depth of 2 influence the expression of target genes *via* other regulators. Figure legend indicates whether genes were upregulated or downregulated in MET relative to PRI tumors; the predicted activation state of the upstream regulators, and the predicted relationships between these and downstream genes and functions.

We then used the Upstream Analysis and Causal Network module of IPA to understand how the abovementioned functions might be regulated in our dataset by activated or inhibited upstream regulators. **Supplementary Data Sheet 6** lists all the predicted activated or inhibited master regulators (z-score >2, <-2) which are hypothesized to control the expression of our dataset molecules either directly or indirectly through other regulators. Of these, *IL1RN* (interleukin 1 receptor antagonist) is predicted to be a significantly inhibited master regulator contributing to the gene expression changes seen in MET vs. primary (PRI+ or PRI) tumors. *IL1RN* acts indirectly on downstream targets distinguishing MET from PRI+ by mediating the activity of intermediary regulators including *TGFB1* (an important TSK/EMT marker) and the inflammatory cytokines *TNF*, *IFNG*, and *IL1* (with high confidence of activation) (**Figure 4A**). This then leads to the upregulation *of VEGFA*, *TIMP1*, *SPP1*, *ITGA5*, and *CEACAM5*, all known to be associated with increased invasiveness (9, 56, 65), and downregulation of various genes including the tumor-suppressor *APC* (APC regulator of WNT signaling pathway). Altogether, this is predicted to increase the neoplasia of tumor cells, migration of tumor and leukocytes, and decrease apoptosis of tumor cell lines (**Figure 4A**). In MET vs. PRI-, *IL1RN* acts directly on downstream targets such as *VEGFA*, *TIMP1*, *SPP1*, and *MMP1* and the stem cell gene *CD44* (**Supplementary Data Sheet 6**).

*VEGFA* (vascular endothelial growth factor A) and *EGF* (epidermal growth factor) are predicted to be significantly activated master regulators driving differential gene expression in MET vs. PRI- (**Supplementary Data Sheet 6**). Both growth factors are known stimulators of uPAR mRNA expression (23). **Figure 4B** demonstrates the VEGFA-mediated upregulation of genes involved in ECM interaction and MMP remodeling (also TSK-specific genes (9)) such as *MMP1*, *MMP10*, *MMP12*, *PLAU*, and *CXCL10* in MET, as well as *FLT1* (encodes VEGFA receptor), which in turn promote metastatic functions such as angiogenesis, growth, migration and invasion, and evasion of apoptosis. **Figure 4C** demonstrates the EGF-mediated upregulation of genes of an overlapping subset of genes as well as *PLAUR*, *VEGFA*, and *KRT19* and a variety of transcription factors in MET. *EGFR* mRNA levels were high in all cohorts, and there was no significant differential expression in any of the tumor comparison or in tumor vs. SES (**Supplementary Data Sheet 2**).

In PRI+ vs. PRI- analysis, *JAG1* (encodes Jagged Canonical Notch Ligand 1) appeared as one of the main activated master regulators (z-score = 2.668, *p* = 6.43E^-10^) predicted to act through *AKT*, *EGFR*, *ERK1/2*, *NOTCH1*, and *TCF7L2* leading to the expression of matrix remodeling genes *MMP1*, *MMP10*, *ICAM1*, and *PLAUR* (**Supplementary Data Sheet 6**), possibly from TSKs sampled from the leading edge of PRI+ tumors.

### 3.4 uPAR Protein Levels Are Significantly Increased in Metastatic cSCC and Correlates With Downregulation of hsa-miR-340-5p and hsa-miR-377-3p

**Figure 5:**
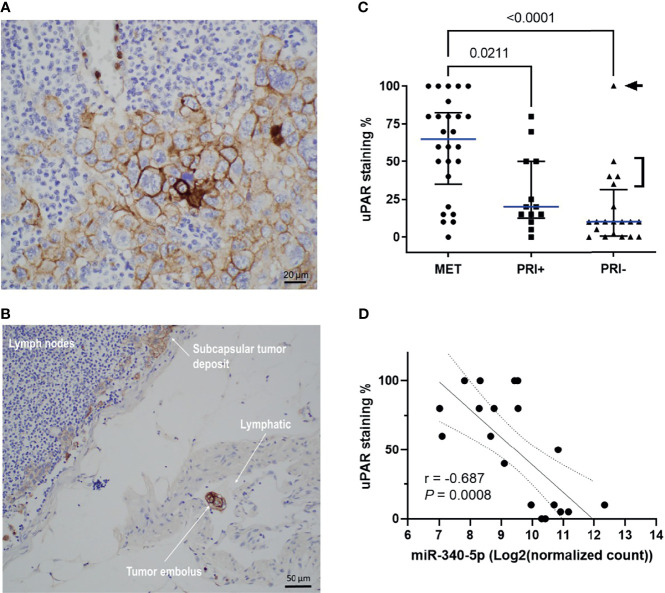
uPAR protein expression is increased on metastatic tumors and correlates with miRNA-340-5p expression. Representative photomicrographs showing uPAR staining in **(A)** a metastatic deposit in the lymph node of Patient 2 and **(B)** a positively stained embolus as well as staining in the subcapsular tumor deposit from the lymphatic of patient 2. **(C)** Scatter plot of uPAR IHC scores of all patient specimens stained (n = 58 total) showing cohort median values (blue line) with interquartile ranges (refer to **Table S3** for individual patient values). *p*-values shown were derived using a Kruskal–Wallis test for multiple comparisons with uncorrected Dunn’s posttest. Arrowhead denotes patient 37 who had a 160 mm diameter × 70 mm thick PRI-. Bracket denotes PRI- tumors with >50-mm diameter and PNI. **(D)** Scatter plot showing relationship between uPAR staining scores and hsa-miR-340-5p normalized gene count (n = 20 pairs; refer to **Table S6**) and show Rho (Spearman’s) correlation, *p*-value, and interquartile range for the correlation.

Given the upregulation of the genes for uPA and its receptor uPAR in metastatic tumors compared to PRI- and SES (see also **Supplementary Image 4**) and their contribution to tumor progression through different pathways and functions, we examined spatially localized uPAR protein levels in an extended cohort of cSCC tumors of the head and neck. **Figure 5A** shows an example of membranous staining typically found in MET specimens (from lymph node deposits). Interestingly, a positively stained tumor embolus was captured in-transit in a lymphatic vessel (**Figure 5B**), highlighting the upregulation of uPAR on invasive and metastatic tumor cells. uPAR was found to be highly tumor-specific, with increased staining in the tumor compartment, particularly at the leading edge of tumors, with absence of staining in SES (**Supplementary Image 6**). Analysis of the staining scores (**Table S3**) found significantly increased uPAR staining in MET tissues compared to PRI+ and PRI- (*p* = 0.0255 and <0.0001, respectively) (**Figure 5C**). The staining intensity was generally higher in PRI+ than in PRI-, but this was not statistically significant (**Figure 5C**) potentially due to the effects of an outlier in the PRI- group with high uPAR staining (Patient 37, **Table S3**). This specimen was characterized to be a highly invasive 160-mm-diameter × 70-mm-thick exophytic primary tumor in the scalp as opposed to other PRI- tumors with less than 20-mm depth of invasion (**Figure 5C**).

To assess the potential regulation of *PLAUR* expression by miRNAs in cSCC, we extracted *PLAUR* targeting miRNAs from the miRDB based on the target prediction score of >75 (high confidence; **Table S4**) and then from a list of experimentally confirmed miRNA-*PLAUR* interactions compiled from miRTARBASE (**Table S5**). Of these lists, only hsa-miR-340-5p and hsa-miR-377-3p from the miRDB list showed a statistically significant differential expression between MET and combined PRI tumor cohorts with both miRNAs being significantly downregulated in MET (**Table S6**). By computing two-tailed Pearson correlation coefficients, the strongest significant negative correlation was found between miR-340-5p and uPAR staining intensity for our dataset (**Figure 5D**). No significant correlation was found between miR-340-5p and *PLAUR* mRNA expression (data not shown). Interestingly, the expression of both miRNAs and uPAR protein was much higher and lower, respectively, in MET04 than the remainder of the cohort (**Table S6**). In contrast, PRI+02, with 100% positivity for uPAR staining, expressed much lower levels of both miRNAs than the other primary tumors.

### 3.5 EGF Enhances cSCC Cell Motility and uPAR Expression

**Figure 6:**
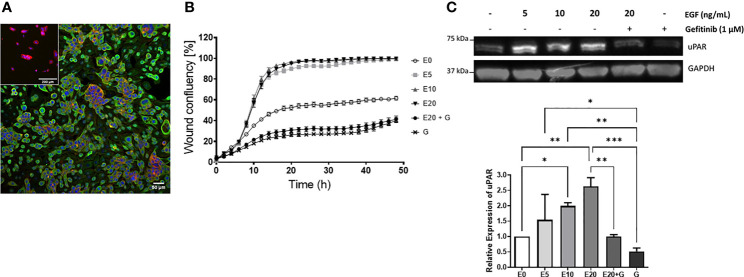
EGF upregulation of cSCC cell line motility and uPAR expression. **(A)** Immunocytochemical image of UW-CSCC2 cells stained with anti-EGFR antibody (green) or anti-mouse IgG negative control (inset) and counterstained with RedDot (blue) and ActinRed 555 (red). **(B)** Representative *in vitro* scratch wound healing assay showing effect of EGF E ± EGFR inhibitor gefitinib G on simple migration of UW-CSCC2 cells. Values shown are mean ± SEM, n = 5; all treatment groups were significantly different from untreated controls *p*-value < 0.05, all gefitinib treatment groups significantly different to EGF only treatment groups *p*-value < 0.001; ordinary one-way ANOVA with Dunnett’s post-test. **(C)** Representative Western blot (right panel) demonstrating UW-CSCC2 uPAR levels in response to 24-h pretreatment with EGF ± gefitinib, at concentrations shown. Panel below: densitometry analysis showing the ratios of uPAR/GAPDH (used as a total protein loading control) for each treatment relative to no EGF control. Significance values are shown with *p-value < 0.05; **p-value < 0.01; ***p-value < 0.001.

Given that uPAR levels were increased on metastatic vs. non metastatic tumors and that EGF was identified as a master regulator leading to *PLAUR* upregulation, we sought to confirm this relationship *in vitro* using a metastatic cSCC cell line derived from a lymph node deposit, UW-CSCC2 (Patient 40, **Table S3**) (52). These cells constitutively express EGFR (**Figure 6A**) (but did not harbor *EGFR* mutations or copy number variations, data not shown) and responded to exogenous 5–20 ng/ml EGF with increased cell migration (**Figure 6B**) and uPAR protein levels (**Figure 6C**) compared to untreated cells. Treatment with the EGFR tyrosine kinase inhibitor gefitinib, even in the presence of 20 ng/ml EGF, significantly inhibited wound closure with respect to both control and EGF-treated cells (**Figure 6B**). uPAR expression levels were also significantly decreased (**Figure 6C**). A second metastatic cSCC cell line (UW-CSCC1; Patient 17, **Table S3**) was found to be similarly affected by EGF/R stimulation and inhibition (data not shown).

## 4 Discussion

**Figure 7:**
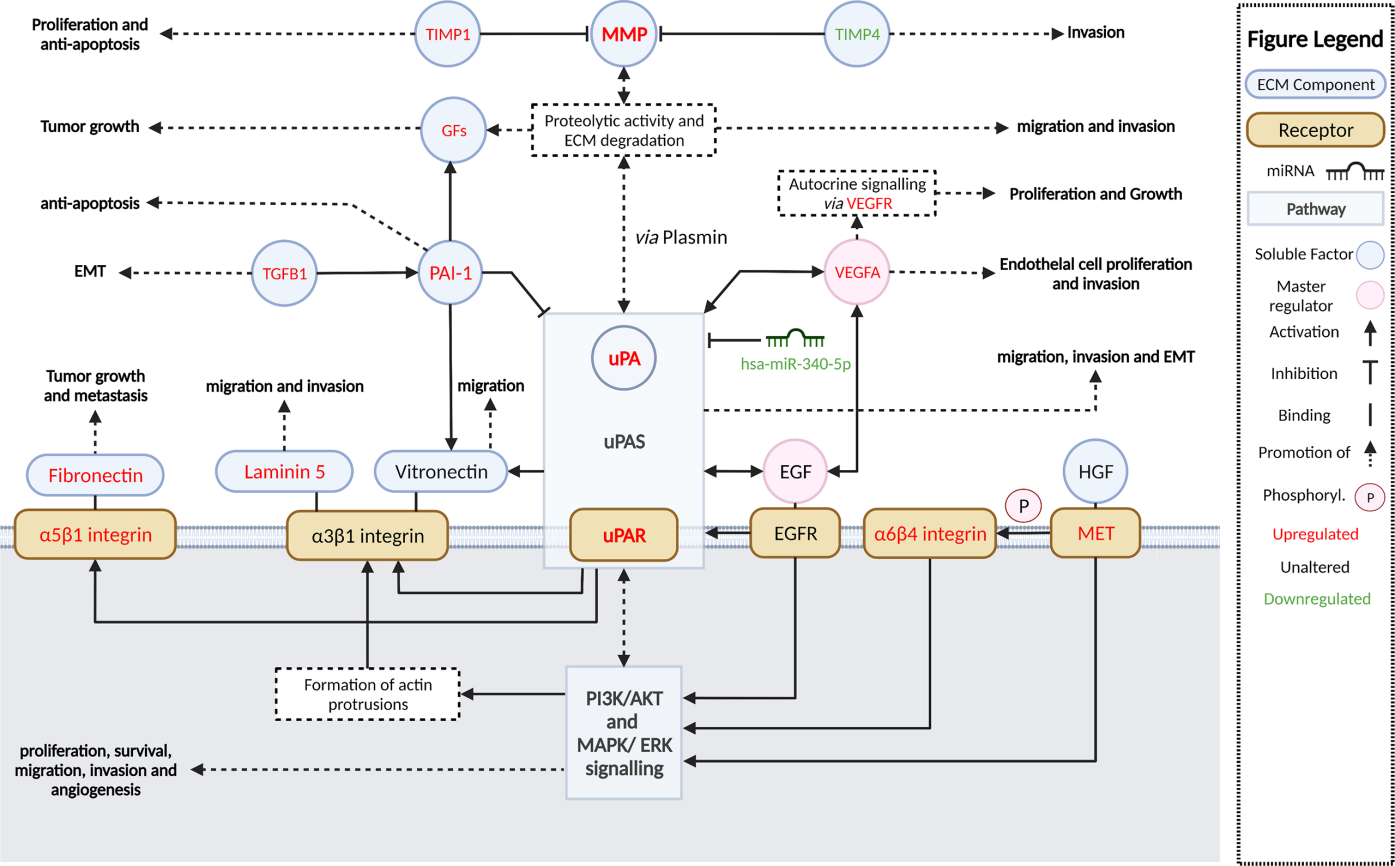
Summary and schematic illustration of key pathways and regulators identified as up- or downregulated in metastatic versus non-metastatic tumors or SES in this study. The urokinase plasminogen activator system (uPAS) plays a central role in remodeling the extracellular matrix (ECM) promoting metastasis. The uPAS exerts its activity by enhanced uPA-mediated conversion of co-localized plasminogen to plasmin and subsequent activation of matrix metalloproteases (MMP). PAI-1 (*SERPINE1*) can inhibit uPA activity but is also upregulated and contributes to cell signaling. MMPs and plasmin cleave and remodel the ECM leading to the release of latent growth factors (GFs) such as EGF, VEGF-A, TGF-β, and HGF (hepatocyte growth factor). By binding to their cognate receptors, EGFR and VEGFR (encoded by *FLT1*), and c-MET, these growth factors in turn act as important upregulators of the uPAS (*via* uPAR) and other downstream effectors, which induce large-scale cellular changes that further promote ECM remodeling, cellular migration, and invasion and, ultimately, metastasis. A few of these growth factor receptors are themselves overexpressed (i.e., *MET*, *FLT1*) and can drive invasion and metastasis regardless of growth factor activation. Aberrant miRNA expression, such as downregulated has-miR-340-5p, is also associated with upregulated uPAR expression. Direct and indirect downstream effectors of the uPAS include vitronectin, focal adhesions *via* integrins and focal adhesion kinase (FAK), the proliferation, and survival pathways MAPK/ERK PI3K/Akt/mTOR and VEGF-A, which facilitate increased protection against apoptosis/anoikis, increased cell proliferation, and EMT and angiogenesis; these are also important for invasion and metastasis. Created with BioRender.com.

Dysregulated activation of extracellular proteolytic networks is strongly linked to mechanisms that enable tumor invasion and metastasis. Our cSCC cohort gene expression and pathway enrichment analyses using various methodologies strongly implicate ECM remodeling and interactions allowing cell motility as among the most significant activated pathways and functions, with stepwise increases in activation from SES to metastatic cSCC. Further, we identified the growth factors EGF and VEGF-A as potential master regulators that concordantly upregulate the expression of ECM remodeling genes encoding uPA/R and MMPs—well-recognized metastasis driver proteases in many cancer types. **Figure 7** summarizes the key molecular alterations we found in MET/PRI+ compared to PRI-/SES which center on the urokinase plasminogen activation system.

The upregulation of plasminogen activation family members and MMPs has been reported in previous studies using squamous cell carcinomas, including those of the skin (33, 35, 51, 54, 57, 66–69) (**Table S1**). It was thus not surprising that *PLAU* and MMP genes were among the highest DEGs in all tumors vs. SES comparisons in our cSCC cohort. However, the quantifiable stepwise increase in expression from SES to PRI- to PRI+/MET has not been previously reported in cSCC derived exclusively from the head and neck. Correspondingly, uPAR protein levels were significantly increased in metastases and this was corroborated by our identification of a significant negative correlation (correlation coefficient <-0.60) between hsa-miR-340-5p and uPAR staining, suggesting that this miRNA plays a role in silencing *PLAUR* at a posttranscriptional level. While several miRNAs have been reported to modulate uPAR expression in a variety of diseases (**Tables S4** **,** **S5**), this particular miRNA–target interaction is a novel finding in cSCC and should be functionally validated in future studies. Interestingly, *SERPINE1* (encodes plasminogen activator inhibitor type 1, a potent inhibitor of uPAR-bound uPA) was also upregulated in all our tumor cohorts (refer to **Supplementary Data Sheet 2**). This is of note because combined upregulated *PLAU* and *SERPINE1* expression is strongly associated with poor cancer outcomes in various other cancers *via* mechanisms that affect cell adhesion, ECM remodeling, and signaling pathways leading to increased cell survival, migration, invasion, and angiogenesis (21, 23, 70–73).

In a study by Ji et al., single-cell RNA sequencing with spatial transcriptomics identified four subpopulations of keratinocytes within primary cSCCs with a specific TSK subpopulation localized to the leading edge (9). The gene signature of the TSKs is uniquely linked to EMT, cellular movement, and extracellular matrix disassembly, suggestive of invasive behavior and that these cells are responsible for metastasis (9). The presence of these subpopulations may explain why our bulk tumor analysis of DEG in PRI+ vs. PRI- could not effectively distinguish the two groups, despite sampling from areas of high tumor cellularity at the leading edge. While bulk tumor analysis represents a limitation of our study, nonetheless we identified significant upregulation of key TSK signature genes, in particular *PLAU*, *MMP1* and *MMP10*, *ITGA5* in MET vs. PRI- and PRI+ vs. PRI-. Interestingly, of these important TSK genes only *MMP10* is included in a 40-gene expression profile test that was recently shown to identify cSCC patients’ risk of metastasis (74). Nevertheless, these genes together with many other genes that were upregulated in MET/PRI+ vs. PRI- have known functions in ECM adhesion and remodeling (e.g., *PLAUR*, *SPP1*, *MMP12*) and/or cell proliferation and motility (e.g., *STAT1* and *CXCL10*). In concordance with the primary cytokine activation signature observed in our Reactome enrichment analyses, Ji *et al.* (9) and others (75) also identified elevated expression of key components of the JAK-STAT pathway (e.g., *STAT1*) and various inflammatory cytokine genes in invasive cSCC. We also identified upregulation of genes encoding the macrophage and CAF ligands, secreted phosphoprotein 1 (*SPP1*), and fibronectin (*FN1*) which have been shown to interact with the TSK receptors integrin subunit beta 1 (*ITGB1*) and subunit alpha 3 or 5 (*ITGA3*, *ITGA5*), respectively, in cSCC (9). This likely reflects the presence of stromal cells in our samples and aberrant tumor–stroma interactions. Notably, a high expression of *ITGB1* and *PLAU* has been shown to be associated with reduced progression-free survival in clinical trials of anti-PD-1 in lung and head and neck mucosal SCC (9, 76). As both genes were upregulated in metastatic cSCC, this suggests that a similar association may occur in cSCC.

We also found that the matrix metalloproteinase inhibitor genes, *TIMP1* and *TIMP4*, were differentially expressed in metastatic versus non-metastatic/SES tissues. Many studies have reported on the elevated expression of *TIMP1* in non-cutaneous head and neck SCC (54–60), but only one of these (56) included any cSCC among their samples. While *TIMP4* has been previously reported to be downregulated in non-cutaneous head and neck SCCs (77), we are the first to report the downregulation of *TIMP4* in MET and PRI+ compared to SES in cSCC. Further, our finding of a positive and negative association with *TIMP1* and *TIMP4* expression, respectively, is in line with a previous study comparing their mRNA and protein expression in normal human brain and malignant gliomas (78). Silencing of *TIMP4 via* hypermethylation of its promoter has been reported in other human cancers (79), with reduced *TIMP4* associated with increased angiogenesis (55, 80–82). Epigenetic regulation of *TIMP4* might also possibly explain *TIMP4* downregulation in metastatic cSCC and should be further explored in future studies.

The activating effects of EGF and VEGF-A on downstream genes including *PLAU/R* highlights the potential for anti-EGFR- and/or anti-VEGF- with anti-uPA/uPAR-targeting approaches for metastatic cSCC. EGFR inhibition as monotherapy for metastatic cSCC has had moderate success (5), even though EGFR is often overexpressed in cSCC, with one study showing an association with EGFR levels and lymph node progression and tumor cell proliferation (83). In our study, *EGFR* mRNA counts were generally equally high across all tumor cohorts and SES suggesting no relationship with tumor status (data not shown) but rather that the presence of high levels of active EGF (and VEGF-A) in the pericellular space of metastatic tumors may be responsible for enhanced stimulation of EGFR-mediated signaling pathways (**Figure 7**). This would contribute to EGFR drug resistance mechanisms through stimulation of compensatory signaling pathways (5) in metastatic cSCC and drive overexpression of downstream targets, including *PLAU/R* and MMP genes, promoting functions linked to cell invasion such as cell motility. The latter was functionally validated in our EGFR-expressing cell line models. Notably, gefitinib significantly inhibited uPAR expression and cell migration, further supporting EGFR tyrosine kinase activation as a mechanism driving uPAR overexpression. Further, as *PLAUR* overexpression was shown to induce gefitinib resistance through the EGFR/p-AKT/surviving signaling pathway in cell models of human lung adenocarcinoma (84), strategies that downregulate *PLAUR* could also be explored to avoid EGFR-targeted resistance mechanisms.

In conclusion, our integrated analysis of the mRNA, miRNA, and uPAR protein expression in a well-characterized spectrum of disease states provides a comprehensive evaluation of the pathways that promote metastasis in cSCC of the head and neck (**Figure 7**). The central role of uPA/R as a biomarker of cSCC metastasis should be further explored using larger cohort studies and with functional studies using metastasis models of cSCC *in vivo*. Combinations of drugs targeting uPA/R and EGFR and/or angiogenesis could be novel therapeutic strategies for metastatic cSCC.

## Data Availability Statement

The original contributions presented in the study are included in the article/**Supplementary Material**. Further inquiries can be directed to the corresponding author.

## Ethics Statement

The studies involving human participants were reviewed and approved by the University of Wollongong Health and Medical Human Research Ethics Committee. Written informed consent for participation was not required for this study in accordance with the national legislation and the institutional requirements.

## Author Contributions

EM, AT, BG, and JP performed and analyzed the gene expression and cell-based experiments and wrote drafts of the manuscripts. SM, JM, and RG compiled and analyzed the clinciopath data and provided specimens. RG performed and analyzed IHC data. BA, JC, and MR conceived and designed the project. MR revised and edited the manuscript and figures. BA and JC edited the finalized the manuscript. BA, JC, RG and MR acquired funding. All authors contributed to the article and approved the submitted version.

## Funding

EM was supported by a CONCERT Translational Cancer Research Centre grant 13/TRC/1-01 to MR. The Sydney Head and Neck Cancer Institute contributed toward NanoString reagents. JP was supported by a NHMRC Ideas Grant APP1181179 awarded to MR, BA, and RG.

## Conflict of Interest

The authors declare that the research was conducted in the absence of any commercial or financial relationships that could be construed as a potential conflict of interest.

## Publisher’s Note

All claims expressed in this article are solely those of the authors and do not necessarily represent those of their affiliated organizations, or those of the publisher, the editors and the reviewers. Any product that may be evaluated in this article, or claim that may be made by its manufacturer, is not guaranteed or endorsed by the publisher.
