# Supplementary material for: Cancer Progression Gene Expression Profiling Identifies the Urokinase Plasminogen Activator Receptor as a Biomarker of Metastasis in Cutaneous Squamous Cell Carcinoma
Source: Front Oncol. 2022 Apr 11;12:835929. doi: 10.3389/fonc.2022.835929 (PMC9035872; doi:10.3389/fonc.2022.835929)
Supplement: Supplementary file 7 [file Image_1.pdf]

A

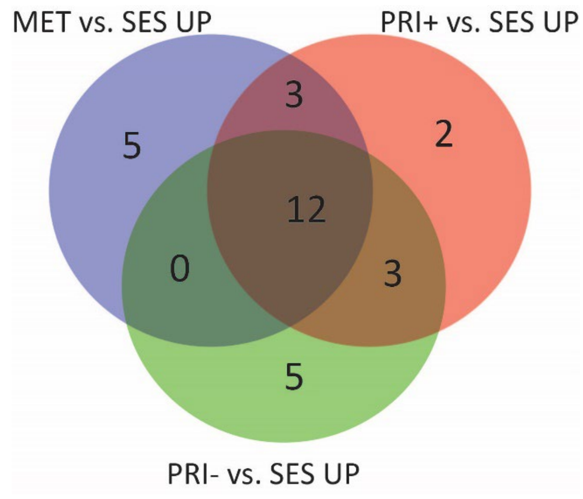

	#	Genes	Key function
Shared up-regulated genes in all tumours and SES	12	*MMP13*	MMP remodeling
		MMP12	MMP remodeling
		CXCL8	Angiogenesis response
		SULF1	Plasma membrane
		INHBA	Cell proliferation
		CXCL11	MMP remodeling
		MMP1	MMP remodeling
		LAMC2	ECM receptor interaction
		LAMB3	ECM receptor interaction
		MMP3	MMP remodeling
		SERPINE1	MMP remodeling
		FN1	Cell motility
Shared up-regulated genes in MET v SES and PRI+ v SES only	3	*PLAU*	MMP remodeling
		KRT19	Epithelial to Mesenchymal Transition
		MMP10	MMP remodeling

B

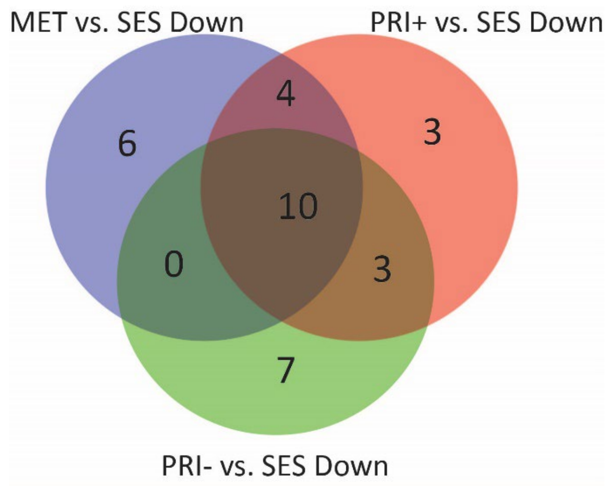

	#	Gene	Key function
Shared down-regulated genes in all tumours and SES	10	*APOD*	Cell adhesion
		LRG1	Positive Regulation of Angiogenesis
		CHI3L1	Positive Regulation of Angiogenesis
		KRT1	ECM Structure
		CLEC3B	ECM Structure
		CD36	Cell Adhesion
		TIMP4	MMP Remodeling
		MYH11	Metastasis Response
		SPINK5	Negative Regulation of Angiogenesis
		PTGDS	Epithelial to Mesenchymal Transition
Shared down-regulated genes in MET v SES and PRI+ v SES only	4	*TF*	HIF1A Signaling, Plasma Membrane
		EMILIN3	ECM structure
		SORD	Cell Motility
		RORA	Angiogenesis Response

C

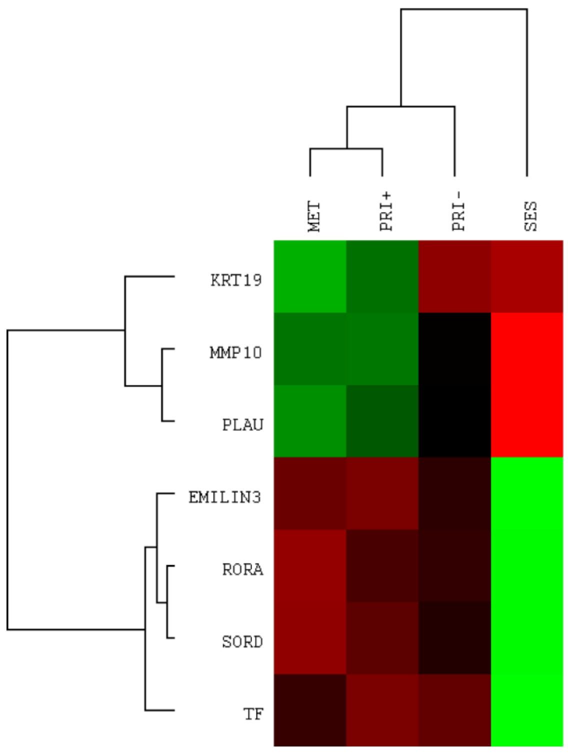

Supplementary Image 1. Venn diagram of a) top 20 up-regulated genes and b) top 20 down-regulated genes between MET vs. SES, PRI+ vs. SES, and PRI- vs. SES (adjP-value < 0.05, Log2 fold change >+1). The number of DEG for each pairwise comparison is indicated in the circles of the Venn diagram. The overlap between the circles shows DEGs that occur in more than one comparison (i.e. are shared). Panels below list the up and down-regulated shared genes between all cohort or MET/PRI+ vs. SES comparisons and their key functions (as annotated by nCounter PanCancer Progression Panel). c) Agglomerative Cluster - Heat Map (using Euclidean distance metric) of selected gene expression of grouped cohort data. Count data has undergone z-score transformation to give equal mean and variance across the samples. Red indicates a higher score, green indicates a lower score.
