# Supplementary material for: Cancer Progression Gene Expression Profiling Identifies the Urokinase Plasminogen Activator Receptor as a Biomarker of Metastasis in Cutaneous Squamous Cell Carcinoma
Source: Front Oncol. 2022 Apr 11;12:835929. doi: 10.3389/fonc.2022.835929 (PMC9035872; doi:10.3389/fonc.2022.835929)
Supplement: Supplementary file 8 [file Image_2.pdf]

A

| Upregulated | | Downregulated | |
|---|---|---|---|
| 1 | *MMP3* | 1 | *SRGN* |
| 2 | *MMP1* | 2 | *CD44* |
| 3 | *CXCL8* | 3 | *TNXB* |
| 4 | *CXCL10* | 4 | *CD34* |
| 5 | *DSC2* | 5 | *RORA* |
| 6 | *CXCL13* | 6 | *CD36* |
| 7 | *MMP10* | | |
| 8 | *STAT1* | | |
| 9 | *ITGA6* | | |
| 10 | *SLPI* | | |
| 11 | *COL4A1* | | |
| 12 | *TNC* | | |
| 13 | *SPP1* | | |
| 14 | *PLAU* | | |
| 15 | *CXCL11* | | |
| 16 | *SLC2A1* | | |
| 17 | *CD24* | | |
| 18 | *SERPINE1* | | |
| 19 | *PLAUR* | | |
| 20 | *LAMC2* | | |
| 21 | *SRGN* | | |
| 22 | *MMP12* | | |
| 23 | *CDKN2A* | | |
| 24 | *PDPN* | | |
| 25 | *HIF1A* | | |
| 26 | *MMP9* | | |
| 27 | *IL1A* | | |

B

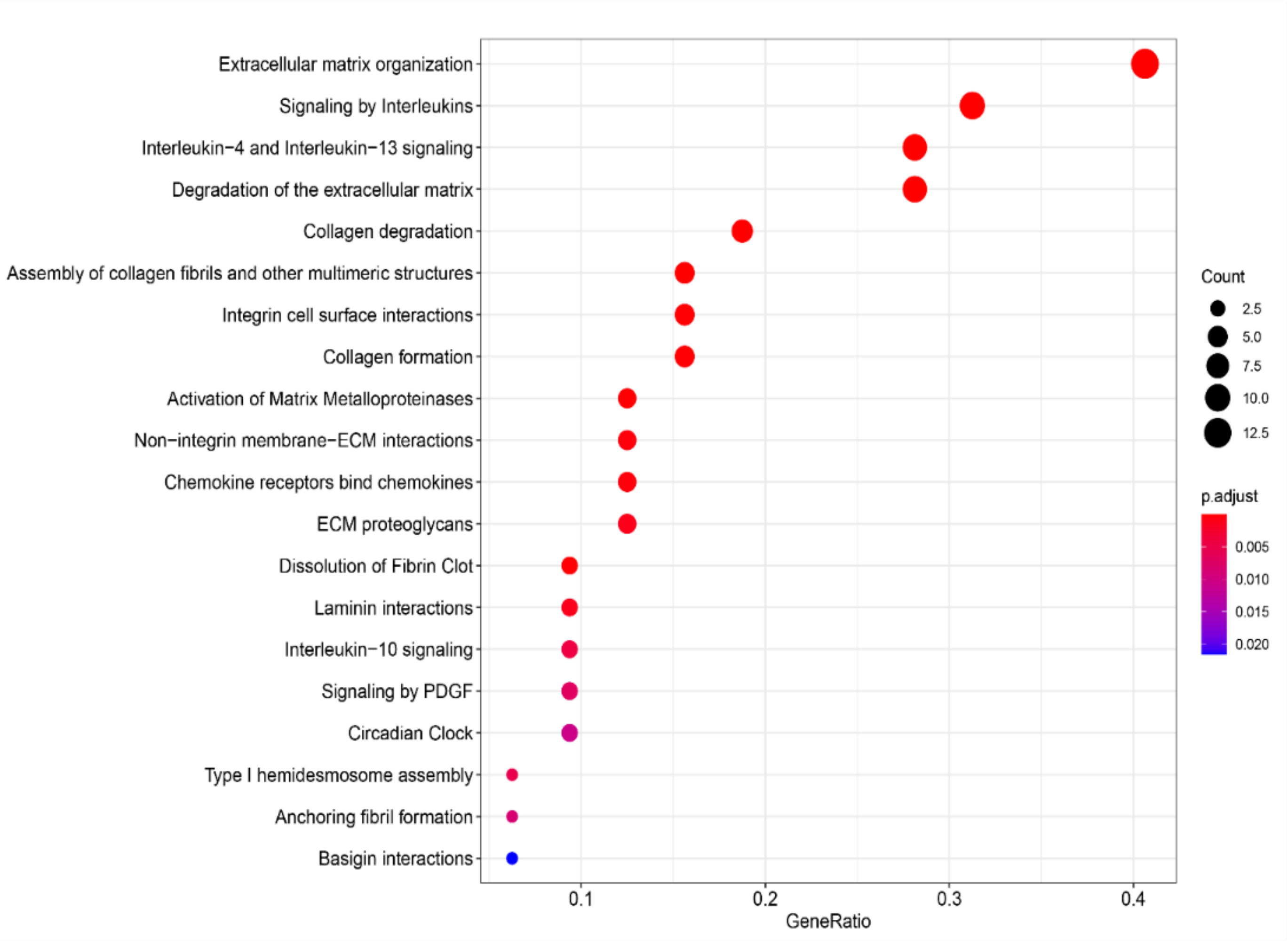


**Supplementary Image 2**. Reactome pathway analysis of combined metastatic vs. normal skin analyses. a) Shared significantly up- and down-regulated genes (log2FC < -0.58, > 0.58; P-value < 0.01) derived from public datasets meta-analyses of metastatic primary tumors vs. normal skin and PRI+ vs. SES (current study). b) Dot plot of top 20 significantly enriched Reactome gene ontology (GO) terms derived using the 33 shared genes shown in a). Plotted in order of gene ratio with the size of the dots representing the number of genes in the gene list associated with the GO term and the color of the dots representing the P-adjusted values.
