# Supplementary material for: Cancer Progression Gene Expression Profiling Identifies the Urokinase Plasminogen Activator Receptor as a Biomarker of Metastasis in Cutaneous Squamous Cell Carcinoma
Source: Front Oncol. 2022 Apr 11;12:835929. doi: 10.3389/fonc.2022.835929 (PMC9035872; doi:10.3389/fonc.2022.835929)
Supplement: Supplementary file 9 [file Image_3.pdf]

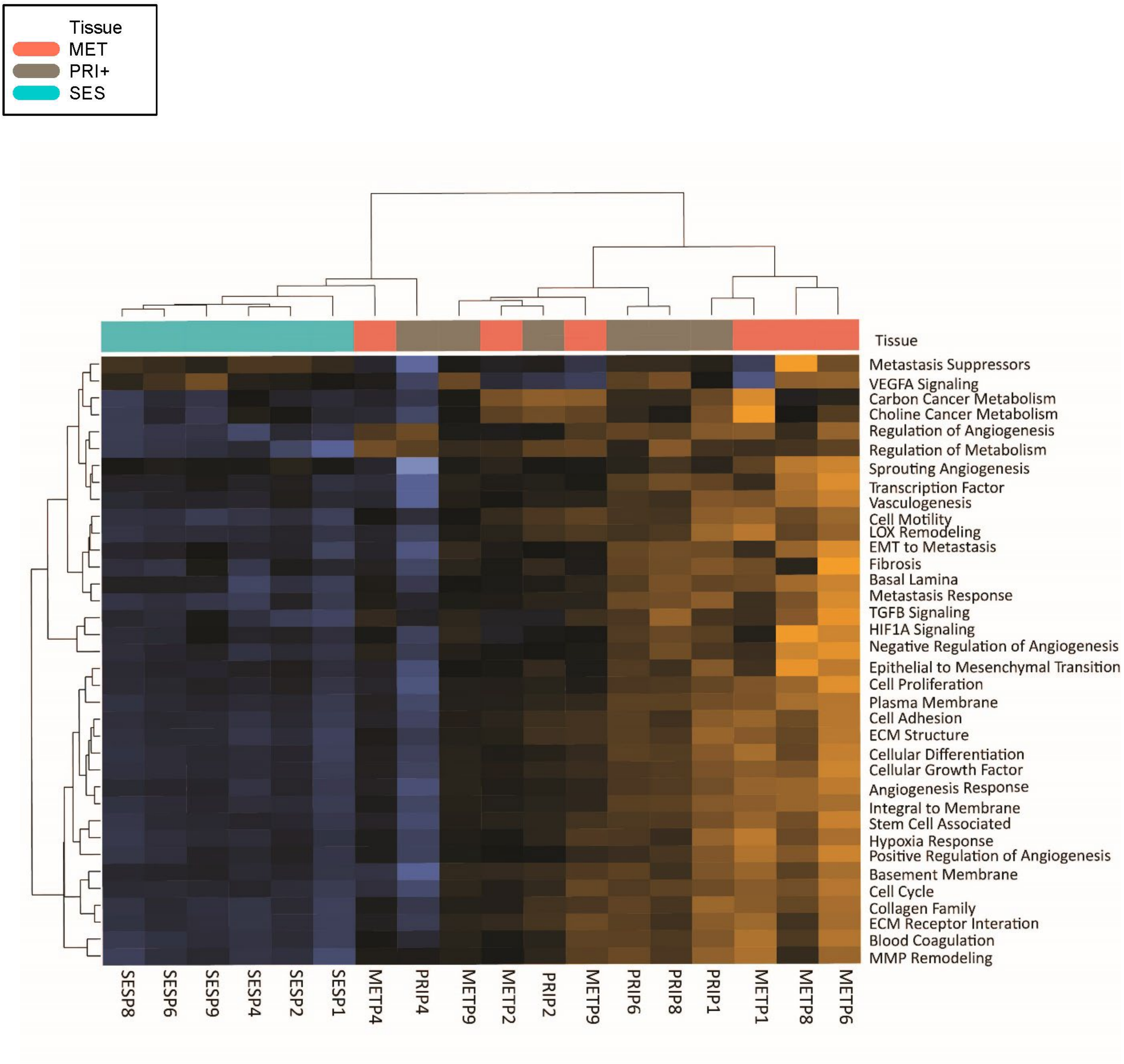

Supplementary Image 3. Intrapatient pathway analyses. Heatmap (unsupervised hierarchal clustering) of global significance scores generated using nSolver Advanced Analysis software 2.0. Orange denotes gene sets whose genes exhibit extensive differential expression with the covariate (SES), blue denotes less differential expression.
