# Supplementary material for: Cancer Progression Gene Expression Profiling Identifies the Urokinase Plasminogen Activator Receptor as a Biomarker of Metastasis in Cutaneous Squamous Cell Carcinoma
Source: Front Oncol. 2022 Apr 11;12:835929. doi: 10.3389/fonc.2022.835929 (PMC9035872; doi:10.3389/fonc.2022.835929)
Supplement: Supplementary file 10 [file Image_4.pdf]

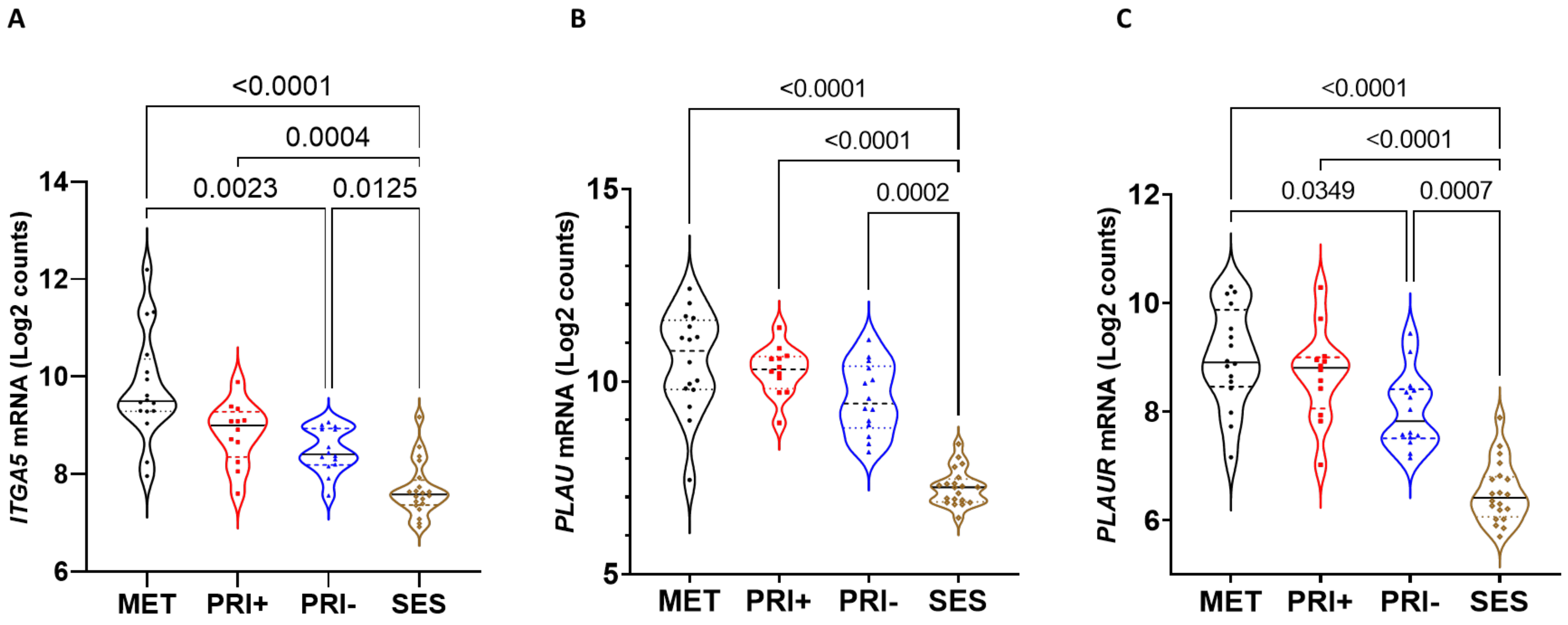

Supplementary Image 4. Violin plots showing a) *ITGA5*, b) *PLAU,* and c) *PLAUR* expression across cohorts. The median value is represented by a horizontal solid line and the quartiles by the dotted lines. The data was analyzed by One-way non-parametric ANOVA (Kruskal-Wallis test) with Dunnet's post hoc test. Significant *P*-values shown above.
