# Supplementary material for: Cancer Progression Gene Expression Profiling Identifies the Urokinase Plasminogen Activator Receptor as a Biomarker of Metastasis in Cutaneous Squamous Cell Carcinoma
Source: Front Oncol. 2022 Apr 11;12:835929. doi: 10.3389/fonc.2022.835929 (PMC9035872; doi:10.3389/fonc.2022.835929)
Supplement: Supplementary file 11 [file Image_5.pdf]

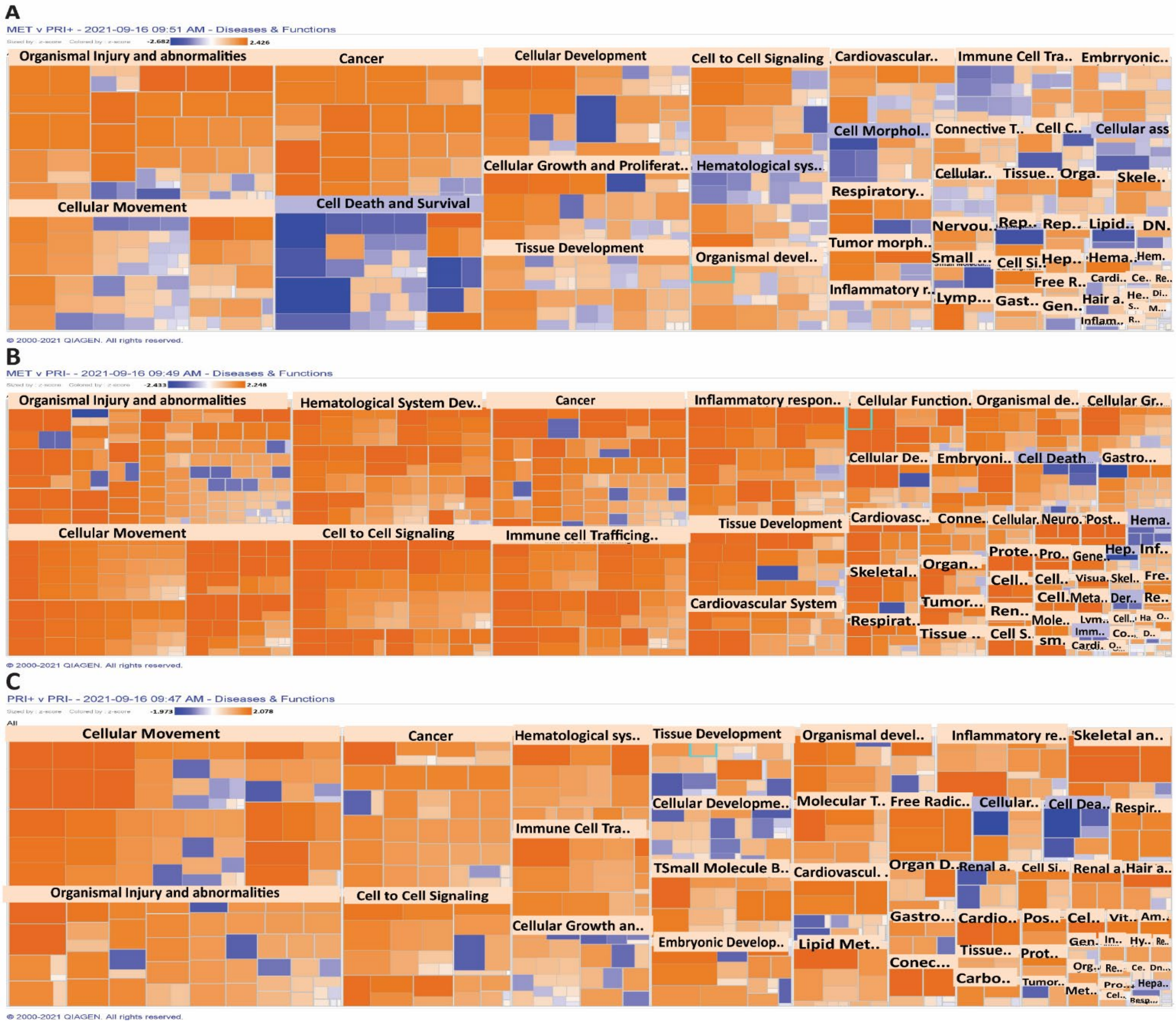

Supplementary Image 5. Top scoring IPA Diseases and Function analysis heatmaps of a) MET vs. PRI+, b) MET vs. PRI-, and c) PRI+ vs. PRI-. The box plots are provided by IPA; each of the many internal boxes represents one annotation, with the box size and the color indicating the z-score. The bar size on top of each heatmap indicates the z-score. Blue indicates inhibition, orange indicates activation.
