# Supplementary material for: Cancer Progression Gene Expression Profiling Identifies the Urokinase Plasminogen Activator Receptor as a Biomarker of Metastasis in Cutaneous Squamous Cell Carcinoma
Source: Front Oncol. 2022 Apr 11;12:835929. doi: 10.3389/fonc.2022.835929 (PMC9035872; doi:10.3389/fonc.2022.835929)
Supplement: Supplementary file 12 [file Image_6.pdf]

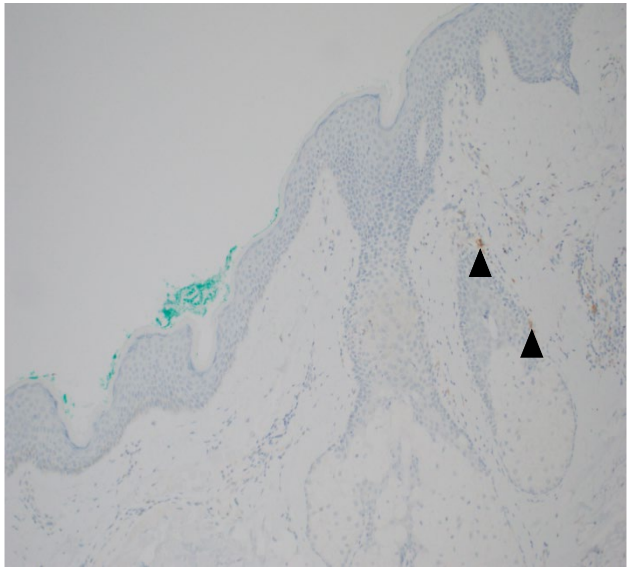

Supplementary Image 6. SES sectioned distantly from any tumors showing lack of uPAR staining in epidermis. Evidence of positive histocytes in dermis (arrowheads). 20x magnification.
