# Supplementary material for: Cancer Progression Gene Expression Profiling Identifies the Urokinase Plasminogen Activator Receptor as a Biomarker of Metastasis in Cutaneous Squamous Cell Carcinoma
Source: Front Oncol. 2022 Apr 11;12:835929. doi: 10.3389/fonc.2022.835929 (PMC9035872; doi:10.3389/fonc.2022.835929)
Supplement: Supplementary file 13 [file Table_1.docx]

**Table S.1 Summary of literature pertaining to expression of the urokinase activation system and ECM adhesion/interaction/remodeling in studies including cutaneous squamaous cell carcinoma (cSCC) clinical samples or cell lines.**

| **Reference** | **GENE** | **Protein** | **Samples** | **Findings** | **GEX in current study** |
| --- | --- | --- | --- | --- | --- |
| [1] | ***LAMB3, LAMC2 and LAMA3*** | Beta, gamma and alpha subunits of Laminin 5 (Laminin332) | 14 squamous cell carcinomas (6 skin, 7 cervix, and 1 vulva) | Most intensive staining in cancer cells esp. at tumour-stroma interface | ↑ significantly in all tumour cohorts v SES |
| [2] | ***LAMC2*** | laminin-5 gamma2 chain | 1) AK  (2) in situ cSCC, , (3) invasive cSCC,  (4) non-sun exposed skin areas | ↑ Significantly in Invasive cSCC v normal skin |  |
|  | ***MMP3*** | MMP 3 |  |  |  |
|  | ***MMP9*** | MMP 9 |  |  |  |
|  | ***MMP12*** | MMP 12 |  |  |  |
|  | ***MMP13*** | MMP 13 |  |  |  |
|  | ***MMP1*** | MMP 1 |  | ↑ Significantly in Invasive cSCC v normal skin and in situ SCC v normal Skin | ↑significantly in MET and PRI+ vs PRI- ↑significantly in all tumours vs. SES |
|  | ***MMP10*** | MMP 10 |  |  |  |
|  | ***PLAU*** | uPA |  | ↑Significantly in AK, in situ and invasive cSCC v normal skin | ↑ significantly in all tumor cohorts v SES |
|  | ***CHI3L1*** | Chitinase 3-like 1 |  | ↑ Significantly in Invasive cSCC v normal skin |  |
|  | ***INHBA*** | Inhibin A |  |  |  |
|  | ***FN1*** | Fibronectin 1 |  |  | ↑ significantly in all tumour cohorts v SES and MET v PRI |
| [3] | ***LAMC2*** | laminin-5 | 10 AKs and 30 primary cSCC (9 matched to AK) | ↑strongly in cSCC | ↑ significantly in all tumor cohorts v SES |
|  | ***MMP1*** | MMP 1 |  |  | ↑significantly in MET and PRI+ vs PRI- ↑significantly in all tumours vs. SES |
|  | ***MMP10*** | MMP 10 |  |  |  |
|  | ***SPP1*** | Osteopontin |  |  | ↑ significantly in all tumor cohorts v SES |
|  | ***PLAU*** | uPA |  |  |  |
|  | ***PLAUR*** | uPAR |  |  |  |
|  | ***MET*** | c-MET |  |  |  |
|  | ***VEGFA*** | vascular endothelial growth factor A |  |  | ↑ significantly in MET v all cohorts |
| [4] | ***MMP1*** | MMP 1 | 3 immunosuppressed organ-transplanted recipients normal skin, 2 AK and invasive cSCC, 5 additional Normal skin from immunocompetent patients | ↑ in non-melanoma skin cancer compared to normal skin. | ↑significantly in MET and PRI+ vs PRI- ↑significantly in all tumours vs. SES |
|  | ***MMP9*** | MMP 9 |  |  | ↑ significantly in all tumor cohorts v SES |
|  | ***NDRG1*** | NDRG1 |  |  |  |
| [5] | ***MMP1*** | MMP 1 | 15 cSCCs, six AKs associated with cSCC and 16 AKs | ↑Significantly in cSCC compared to AK and healthy skin | ↑significantly in MET and PRI+ vs PRI- ↑significantly in all tumours vs. SES |
|  | ***MMP3*** | MMP 3 |  |  | ↑ significantly in all tumor cohorts v SES |
|  | ***MMP2*** | MMP 2 |  |  | Not among the DEG in our study |
| [6] | ***MMP10*** | MMP 10 | cSCCs (n = 9) and unmatched healthy skin samples (n = 7)(a combination of SES and non SES). 18 healthy donors and 28 patients with primary cSCC. | ↑ in cSCC compared to healthy skin | ↑significantly in MET and PRI+ vs PRI- ↑significantly in all tumours vs. SES |
|  | ***ITGA6*** | Integrin Subunit Alpha 6 |  |  | ↑ significantly in all tumor cohorts v SES |
|  | ***ITGB1*** | Integrin Subunit Beta 1 |  |  |  |
|  | ***CXCl11*** | Chemokine 11 |  |  |  |
| [7] | ***MMP13*** | MMP 13 | T1 stage tumors from the larynx, tongue, and skin or mucosa of the face, cheek, lip, nose, and ear were studied | ↑ in highly invasive tumors | ↑ significantly in all tumor cohorts v SES |
|  | ***TIMP1*** | metallopeptidase inhibitor 1 |  |  | ↑ in MET vs SES and MET vs PRI- |
| [8] | ***MMP13*** | MMP 13 | Malignant epidermal tumors: BCC n = 17 cSCC n = 8, and Paget's disease n =8 | collagenase-3 is associated with tumor invasion | ↑ significantly in all tumor cohorts v SES |
|  |  |  |  |  |  |
| [9] | ***ITGA5*** | Integrin Subunit Alpha 5 | 10 human skin SCCs and matched normals | TSKs expressed classic EMT markers such as ITGA5 | ↑ Significantly in MET vs all samples . ↑ Significantly in PRI+ vs SES |
|  | ***PLAU*** | uPA |  | High expression of PLAU and ITGB1 correlated with significantly reduced progression-free survival | ↑ significantly in all tumor cohorts v SES |
|  | ***ITGB1*** | Integrin Subunit Beta 1 |  |  |  |
| [10] | ***PLAU*** | uPA | 10 normal skin, 50 cSCC, 32 adjacent normal vulva, 41 vSCC, 16 normal buccal mucosa, and 49 HNSCC | uPA-expressing macrophages regulate C3-independent C5a generation,lead to immunosuppressive tumor immune microenvironments |  |
| [11] | ***SERPINE1*** | Plasminogen activator inhibitor type 1 (PAI-I) | 20 BCC and 10 SCC. 3 AK | expressed in metastasizing SCC, but not nonmetastasizing BCC | ↑ significantly in all tumor cohorts v SES |
|  | ***PLAU*** | uPA |  |  |  |
| [12] | ***PLAUR*** | uPAR | squamous cell (n = 7) and basal cell (n= 7) carcinomas of the skin | uPAR and uPA are coexpressed in SCC cells at the invasive front but not BCC |  |
| [13] | ***PLAU*** | uPA | uPAR+ and uPAR_ mice with injected skin carcinogen | uPAR is required for efficient skin tumor Onset and growth in mice |  |
| [14] | ***PLAUR*** | uPAR | 21 cases of NMSCs and five normal skin tissue specimens | ↑ expression of (u-PA) in cSCC compared to Normal skin |  |
| [15] | ***IL1A*** | Hematopoietin-1 | Human epidermal cancer cell line | UVB induces the expression of uPA and uPAR |  |
| [16] | ***COL7A1*** | Collagen Type VII Alpha 1 Chain | 10 SES, 10 AKs, and 10 cSCCs (all fresh frozen) | ↑ in cSCC vs SES |  |
| [17] | ***FN1*** | Fibronectin 1 | 12 individually matched tumor and non-tumor skin samples (SES | ↑ in cSCC vs SES | ↑Significantly in MET vs PRI- and MET vs SES |
| [18] | ***COL5A2*** | Collagen alpha-2(V) | Human cSCC cell cultures and Human cSCC tumor and normal tissue samples | ↑ in cSCC vs SES | ↑ significantly in all tumour cohorts v SES and MET v PRI |
|  | ***Col1A2*** | Collagen Type I Alpha 2 Chain |  |  | ↑significantly in all patient samples compared to SES |
|  | ***COL3A1*** | Collagen Type III Alpha 1 Chain |  |  |  |
|  | ***MMP1*** | MMP 1 |  |  |  |
| [19] | ***TNFRSF12A*** | TNF Receptor Superfamily Member 12A | 56 formalin-fixed paraffin-embedded (FFPE) cutaneous lesions (n = 8 actinic keratosis, n = 30 carcinoma in situ [CIS], n = 18 invasive) and 43 FFPE ocular surface lesions (n = 2 conjunctival/corneal intraepithelial neoplasia, n = 20 CIS, n = 21 invasive) | ↑ significantly in cSCCs relative to precursors | ↑significantly in MET and PRI+ vs PRI- ↑significantly in all tumours vs. SES |
|  | ***MMP3*** | MMP 3 |  |  | ↑significantly in all patient samples compared to SES |
|  | ***MMP9*** | MMP 9 |  |  |  |
|  | ***LAMC2*** | laminin-5 gamma2 chain |  |  |  |
|  | ***SULF1*** | Extracellular Sulfatase Sulf-1 |  |  |  |
| [20] | ***MMP1*** | MMP 1 | Tissue samples of normal nonsun- exposed skin (n=6), normal sun-exposed skin (n=6), AK (n=6),and cSCC (n=6) - combined fresh and formalin fixed samples | ↑ significantly in cSCCs and AK relative to normal nonsun- exposed skin and normal sun-exposed skin | ↑significantly in all patient samples compared to SES |
|  | ***MMP110*** | MMP 10 |  |  | ↑significantly in MET and PRI+ vs PRI- ↑significantly in all tumours vs. SES |
|  | ***MMP13*** | MMP 13 |  |  |  |
|  |  |  |  |  | ↑ significantly in all tumour cohorts vs. SES |

1. Pyke, C., et al., *Laminin-5 is a marker of invading cancer cells in some human carcinomas and is coexpressed with the receptor for urokinase plasminogen activator in budding cancer cells in colon adenocarcinomas.* Cancer Res, 1995. **55**(18): p. 4132-4139.

2. Mitsui, H., et al., *Gene expression profiling of the leading edge of cutaneous squamous cell carcinoma: IL-24-driven MMP-7.* J Invest Dermatol, 2014. **134**(5): p. 1418-1427.

3. Lambert, S.R., et al., *Key differences identified between actinic keratosis and cutaneous squamous cell carcinoma by transcriptome profiling.* Br J Cancer, 2014. **110**(2): p. 520-9.

4. Nindl, I., et al., *Identification of differentially expressed genes in cutaneous squamous cell carcinoma by microarray expression profiling.* Mol Cancer, 2006. **5**(1): p. 30.

5. Tsukifuji, R., et al., *Expression of matrix metalloproteinase-1, -2 and -3 in squamous cell carcinoma and actinic keratosis.* Br J Cancer, 1999. **80**(7): p. 1087-91.

6. Das Mahapatra, K., et al., *A comprehensive analysis of coding and non-coding transcriptomic changes in cutaneous squamous cell carcinoma.* Sci Rep, 2020. **10**(1): p. 3637.

7. Culhaci, N., et al., *Elevated expression of MMP-13 and TIMP-1 in head and neck squamous cell carcinomas may reflect increased tumor invasiveness.* BMC cancer, 2004. **4**(1): p. 42.

8. Airola, K., et al., *Human collagenase-3 is expressed in malignant squamous epithelium of the skin.* J Invest Dermatol, 1997. **109**(2): p. 225-31.

9. Ji, A.L., et al., *Multimodal Analysis of Composition and Spatial Architecture in Human Squamous Cell Carcinoma.* Cell, 2020. **182**(2): p. 497-514 e22.

10. Medler, T.R., et al., *Complement C5a fosters squamous carcinogenesis and limits T cell response to chemotherapy.* Cancer Cell, 2018. **34**(4): p. 561-578. e6.

11. Sappino, A., et al., *Differential protease expression by cutaneous squamous and basal cell carcinomas.* The Journal of clinical investigation, 1991. **88**(4): p. 1073-1079.

12. Rømer, J., et al., *Cancer cell expression of urokinase-type plasminogen activator receptor mRNA in squamous cell carcinomas of the skin.* Journal of investigative dermatology, 2001. **116**(3): p. 353-358.

13. Mazzieri, R., et al., *Urokinase receptor promotes skin tumor formation by preventing epithelial cell activation of Notch1.* Cancer research, 2015. **75**(22): p. 4895-4909.

14. Jee, B.A., et al., *Molecular classification of basal cell carcinoma of skin by gene expression profiling.* Mol Carcinog, 2015. **54**(12): p. 1605-12.

15. Marschall, C., et al., *UVB increases urokinase-type plasminogen activator receptor (uPAR) expression.* J Invest Dermatol, 1999. **113**(1): p. 69-76.

16. Garcia-Diez, I., et al., *Transcriptome and cytogenetic profiling analysis of matched in situ/invasive cutaneous squamous cell carcinomas from immunocompetent patients.* Genes Chromosomes Cancer, 2019. **58**(3): p. 164-174.

17. Hudson, L.G., et al., *Microarray analysis of cutaneous squamous cell carcinomas reveals enhanced expression of epidermal differentiation complex genes.* Mol Carcinog, 2010. **49**(7): p. 619-29.

18. Dooley, T.P., et al., *Biomarkers of human cutaneous squamous cell carcinoma from tissues and cell lines identified by DNA microarrays and qRT-PCR.* Biochem Biophys Res Commun, 2003. **306**(4): p. 1026-36.

19. Lazo de la Vega, L., et al., *Invasive squamous cell carcinomas and precursor lesions on UV-exposed epithelia demonstrate concordant genomic complexity in driver genes.* Mod Pathol, 2020.

20. Zou, B., et al., *Identification of key candidate genes and pathways in oral squamous cell carcinoma by integrated Bioinformatics analysis.* Exp Ther Med, 2019. **17**(5): p. 4089-4099.
