# Supplementary material for: Cancer Progression Gene Expression Profiling Identifies the Urokinase Plasminogen Activator Receptor as a Biomarker of Metastasis in Cutaneous Squamous Cell Carcinoma
Source: Front Oncol. 2022 Apr 11;12:835929. doi: 10.3389/fonc.2022.835929 (PMC9035872; doi:10.3389/fonc.2022.835929)
Supplement: Supplementary file 14 [file Table_2.docx]

**Table S.2. Datasets used for meta-analysis^a^**

| **GEO-ID** | **Organism** | **Platform** | **Source** | **SES** | **cSCC Invasive** |
| --- | --- | --- | --- | --- | --- |
| [GSE45164](https://www.ncbi.nlm.nih.gov/geo/query/acc.cgi?acc=GSE45164)  [1] | Human | GPL571 [HG-U133A_2] | Surgical excision | 3 | 10 |
| GSE53462  [2] | Human | GPL10558 | Patients | 5 (Sun exposed regions) | 5 (non-melanoma) |
| [GSE108010](https://www.ncbi.nlm.nih.gov/geo/query/acc.cgi?acc=GSE108010)  [3] | Human | GPL16686 | Skin Biopsy | 10 | 10 |

^a^Exclusion criteria for datasets or specimens were:

- Cell-lines related datasets
- Datasets collected from immunocompromised patients (due to organ transplant or diseases other than cancer)
- Specimens with perineural invasion (PNI)
- Specimens sourced from non-sun exposed regions
- Specimens of SCC *in situ* or actinic keratosis (non-invasive/AK)
- Specimens from cutaneous basal cell carcinoma (BCC) or melanocytes, cutaneous neoplasm or melanoma skin
- Datasets of oral squamous cell carcinoma

Note: Specimens having no information about sun exposure but were within sun exposed regions were considered as sun-exposed

1. Brooks, Y.S., et al., *Multifactorial ERbeta and NOTCH1 control of squamous differentiation and cancer.* J Clin Invest, 2014. **124**(5): p. 2260-76.

2. Jee, B.A., et al., *Molecular classification of basal cell carcinoma of skin by gene expression profiling.* Mol Carcinog, 2015. **54**(12): p. 1605-12.

3. Garcia-Diez, I., et al., *Transcriptome and cytogenetic profiling analysis of matched in situ/invasive cutaneous squamous cell carcinomas from immunocompetent patients.* Genes Chromosomes Cancer, 2019. **58**(3): p. 164-174.
