# Supplementary material for: Cancer Progression Gene Expression Profiling Identifies the Urokinase Plasminogen Activator Receptor as a Biomarker of Metastasis in Cutaneous Squamous Cell Carcinoma
Source: Front Oncol. 2022 Apr 11;12:835929. doi: 10.3389/fonc.2022.835929 (PMC9035872; doi:10.3389/fonc.2022.835929)
Supplement: Supplementary file 15 [file Table_3.docx]

**Table S.3 uPAR staining by immunohistochemistry (IHC) (n = 58 specimens) in total**

| **Patient#** | **Specimen ID** | **Tumor IHC score** | **Primary Antibody source** |  | **Patient#** | **Specimen ID** | **Tumor IHC score** | **Primary Antibody source** |
| --- | --- | --- | --- | --- | --- | --- | --- | --- |
| P1 | MET01 | 40% | DAKO |  | P1 | PRI+01 | 15% | DAKO |
| P2 | MET02 | 100% | DAKO |  | P2 | PRI+02 | 50% | Sino |
| P3 | MET03 | 80% | DAKO |  | P3 | PRI+03 | 10% | DAKO |
| P4 | MET04 | 0% | Sino |  | P4 | PRI+04 | 5% | DAKO |
| P5 | MET05 | 70% | Sino |  | P6 | PRI+06 | 15% | Sino |
| P7 | MET07 | 50% | DAKO |  | P7 | PRI+07 | 20% | Sino |
| P8 | MET08 | 15% | Sino |  | P8 | PRI+08 | 25% | Sino |
| P9 | MET9 | 10% | Sino |  | P9 | PRI+09 | 20% | Sino |
| P10 | MET10 | 10% | Sino |  | P10 | PRI+10 | 80% | Sino |
| P11 | MET11 | 90% | Sino |  | P18 | PRI+11 | 70% | Sino |
| P12 | MET12 | 100% | Sino |  | P19 | PRI+12 | 0 | Sino |
| P13 | MET13 | 80% | Sino |  | P20 | PRI+13 | 50% | Sino |
| P14 | MET14 | 80% | DAKO |  | P21 | PRI+14 | 15% | Sino |
| P15 | MET15 | 100% | DAKO |  |  |  |  |  |
| P16 | MET16 | 100% | DAKO |  | P22 | PRI-01 | 10% | DAKO |
| P17 | MET17 | 60% | Sino |  | P23 | PRI-02 | 40% | DAKO |
| P18 | MET18 | 20% | Sino |  | P24 | PRI-03 | 20% | DAKO |
| P20 | MET19 | 70% | Sino |  | P25 | PRI-04 | 0 | DAKO |
| P21 | MET20 | 50% | Sino |  | P26 | PRI-05 | 10% | Sino |
| P38 | MET21 | 15% | Sino |  | P27 | PRI-06 | 10% | DAKO |
| P40 | MET22 | 50% | Sino |  | P28 | PRI-07 | 2% | Sino |
| P43 | MET23 | 60% | Sino |  | P29 | PRI-08 | 0 | DAKO |
| P44 | MET24 | 60% | Sino |  | P30 | PRI-09 | 35% | DAKO |
| P45 | MET25 | 80% | DAKO |  | P31 | PRI-10 | 50% | DAKO |
| P46 | MET26 | 80% | Sino |  | P32 | PRI-11 | 0 | DAKO |
| P47 | MET27 | 100% | Sino |  | P33 | PRI-12 | 5% | DAKO |
|  |  |  |  |  | P34 | PRI-13 | 0 | DAKO |
|  |  |  |  |  | P35 | PRI-14 | 10% | DAKO |
|  |  |  |  |  | P36 | PRI-15 | 0 | DAKO |
|  |  |  |  |  | P37 | PRI-16 | 100% | DAKO |
|  |  |  |  |  | P39 | PRI-17 | 40% | DAKO |
|  |  |  |  |  | P41 | PRI-18 | 10% | DAKO |
|  |  |  |  |  | P42 | PRI-19 | 10% | DAKO |
|  |  |  |  |  | P48 | PRI-20 | 10% | Sino |
