# Supplementary material for: Cancer Progression Gene Expression Profiling Identifies the Urokinase Plasminogen Activator Receptor as a Biomarker of Metastasis in Cutaneous Squamous Cell Carcinoma
Source: Front Oncol. 2022 Apr 11;12:835929. doi: 10.3389/fonc.2022.835929 (PMC9035872; doi:10.3389/fonc.2022.835929)
Supplement: Supplementary file 16 [file Table_4.docx]

**Table S.4 miRDB based targets of *PLAUR***

| **Target Score** | **miRNA Name** | **Gene Symbol** |
| --- | --- | --- |
| 95 | [hsa-miR-4517](http://mirdb.org/cgi-bin/mature_mir.cgi?name=hsa-miR-4517) | PLAUR |
| 94 | [hsa-miR-2054](http://mirdb.org/cgi-bin/mature_mir.cgi?name=hsa-miR-2054) | PLAUR |
| 92 | [hsa-miR-942-5p](http://mirdb.org/cgi-bin/mature_mir.cgi?name=hsa-miR-942-5p) | PLAUR |
| 91 | [hsa-miR-5692a](http://mirdb.org/cgi-bin/mature_mir.cgi?name=hsa-miR-5692a) | PLAUR |
| 90 | [hsa-miR-3152-3p](http://mirdb.org/cgi-bin/mature_mir.cgi?name=hsa-miR-3152-3p) | PLAUR |
| 90 | [hsa-miR-3163](http://mirdb.org/cgi-bin/mature_mir.cgi?name=hsa-miR-3163) | PLAUR |
| 89 | [hsa-miR-340-5p](http://mirdb.org/cgi-bin/mature_mir.cgi?name=hsa-miR-340-5p) | PLAUR |
| 88 | [hsa-miR-5590-3p](http://mirdb.org/cgi-bin/mature_mir.cgi?name=hsa-miR-5590-3p) | PLAUR |
| 88 | [hsa-miR-196a-1-3p](http://mirdb.org/cgi-bin/mature_mir.cgi?name=hsa-miR-196a-1-3p) | PLAUR |
| 88 | [hsa-miR-142-5p](http://mirdb.org/cgi-bin/mature_mir.cgi?name=hsa-miR-142-5p) | PLAUR |
| 87 | [hsa-miR-12115](http://mirdb.org/cgi-bin/mature_mir.cgi?name=hsa-miR-12115) | PLAUR |
| 83 | [hsa-miR-195-3p](http://mirdb.org/cgi-bin/mature_mir.cgi?name=hsa-miR-195-3p) | PLAUR |
| 83 | [hsa-miR-16-2-3p](http://mirdb.org/cgi-bin/mature_mir.cgi?name=hsa-miR-16-2-3p) | PLAUR |
| 80 | [hsa-miR-2052](http://mirdb.org/cgi-bin/mature_mir.cgi?name=hsa-miR-2052) | PLAUR |
| 80 | [hsa-miR-5008-5p](http://mirdb.org/cgi-bin/mature_mir.cgi?name=hsa-miR-5008-5p) | PLAUR |
| 80 | [hsa-miR-4283](http://mirdb.org/cgi-bin/mature_mir.cgi?name=hsa-miR-4283) | PLAUR |
| 79 | [hsa-miR-377-3p](http://mirdb.org/cgi-bin/mature_mir.cgi?name=hsa-miR-377-3p) | PLAUR |
| 77 | [hsa-miR-561-3p](http://mirdb.org/cgi-bin/mature_mir.cgi?name=hsa-miR-561-3p) | PLAUR |
| 76 | [hsa-miR-3670](http://mirdb.org/cgi-bin/mature_mir.cgi?name=hsa-miR-3670) | PLAUR |
