# Supplementary material for: Cancer Progression Gene Expression Profiling Identifies the Urokinase Plasminogen Activator Receptor as a Biomarker of Metastasis in Cutaneous Squamous Cell Carcinoma
Source: Front Oncol. 2022 Apr 11;12:835929. doi: 10.3389/fonc.2022.835929 (PMC9035872; doi:10.3389/fonc.2022.835929)
Supplement: Supplementary file 17 [file Table_5.docx]

| **Table S.5 miRtarbase (experimentally validated interactions) based miRNA targets for *PLAUR*** | | | | | | | | | | |
| --- | --- | --- | --- | --- | --- | --- | --- | --- | --- | --- |
| **miRNA** | **Gene** | **Reporter assay** | **Western Blot** | **qPCR** | **Microarry** | **NGS** | **pSILAC** | **Others** | **Sum** | **Papers** |
| hsa-miR-204-5p | PLAUR |  |  |  | Y |  |  |  | 1 | 1 |
| hsa-miR-155-5p | PLAUR |  |  |  |  |  |  |  | 1 | 1 |
| hsa-miR-16-5p | PLAUR |  |  |  |  |  |  | Y | 1 | 1 |
| hsa-miR-146a-5p | PLAUR | Y | Y | Y |  |  |  |  | 3 | 1 |
| hsa-miR-335-5p | PLAUR | Y | Y | Y |  |  |  |  | 3 | 1 |
| hsa-miR-622 | PLAUR | Y | Y | Y |  |  |  |  | 3 | 1 |
