# Supplementary material for: Cancer Progression Gene Expression Profiling Identifies the Urokinase Plasminogen Activator Receptor as a Biomarker of Metastasis in Cutaneous Squamous Cell Carcinoma
Source: Front Oncol. 2022 Apr 11;12:835929. doi: 10.3389/fonc.2022.835929 (PMC9035872; doi:10.3389/fonc.2022.835929)
Supplement: Supplementary file 18 [file Table_6.docx]

**Table S.6**. **Differential expression of selected miRNAs in MET vs. PRI.** List of samples with available miRNA data with normalized read counts for miRNAs and IHC score for each patient shown. For the purposes of this analysis the PRI+ and PRI- specimens were collated.

|  | | Normalized read counts | | uPAR IHC |
| --- | --- | --- | --- | --- |
| Patient# | Specimen Type & ID | hsa-miR-377-3p | hsa-miR-340-5p | Score |
| P2 | PRI+02 | 16.8 | 738.1 | 100% |
| P3 | PRI+03 | 74.2 | 5198.7 | 10% |
| P4 | PRI+04 | 17.9 | 1924.9 | 5% |
| P29 | PRI-08 | 6.3 | 1371.1 | 0% |
| P31 | PRI-10 | 23.2 | 1814.5 | 50% |
| P32 | PRI-11 | 12.8 | 1368.3 | 0% |
| P34 | PRI-13 | 34.0 | 1262.8 | 0% |
| P42 | PRI-19 | 24.0 | 1664.3 | 10% |
| P48 | PRI-20 | 8.1 | 995.4 | 10% |
| P49 | PRI+15 | 84.6 | 1521.5 | N/A |
| P50 | PRI-21 | 67.7 | 1739.9 | N/A |
|  | **mean** | 33.606 | 1781.77 |  |
| P1 | MET01 | 3.5 | 549.0 | 40% |
| P2 | MET02 | 0.0 | 319.4 | 100% |
| P3 | MET03 | 0.0 | 436.5 | 80% |
| P4 | MET04 | 18.1 | 2290.3 | 5% |
| P13 | MET13 | 0.0 | 312.0 | 80% |
| P14 | MET14 | 0.0 | 129.6 | 80% |
| P15 | MET15 | 0.0 | 688.7 | 100% |
| P43 | MET23 | 0.0 | 404.8 | 60% |
| P44 | MET24 | 0.0 | 136.7 | 60% |
| P46 | MET26 | 0.0 | 745.3 | 80% |
| P47 | MET27 | 1.8 | 226.3 | 100% |
| P49 | MET28 | 1.1 | 1090.6 | N/A |
|  | **mean** | 3.75 | 33.61 |  |
|  | log2FC MET v PRI | -3.39 | -1.34 | 1.65 |
|  | P value | 3.42E-05 | 2.76E-03 | 0.0001 |
|  | Padj value | 2.76E-04 | 8.23E-03 | 0.6 |
